# Gellan Gum Modification and Structural Regulation for Food Applications: Delivery Systems and Digestive Behavior

**DOI:** 10.3390/foods15183269

**Published:** 2026-09-16

**Authors:** Yong Zhou, Shiyi Chen, Yan Jiang, Afira Nayab, Ling Sun

**Affiliations:** School of Food Science and Engineering, Jiangsu University, Zhenjiang 212013, China; 15984555767@163.com (Y.Z.); 18852875229@163.com (S.C.); 18361867873@163.com (Y.J.); afiranayab@outlook.com (A.N.)

**Keywords:** gellan gum, modification, structural regulation, food hydrocolloid, food applications, digestive behavior

## Abstract

Gellan gum (GG) is an anionic microbial polysaccharide widely used in foods because of its efficient gelation, thermal stability, and ion-responsive structuring. However, the contrasting limitations of high- and low-acyl GG, together with their sensitivity to molecular weight, ions, pH, thermal history, and food-matrix composition, restrict their performance in complex products. This review distinguishes four intervention levels of GG modification and structural regulation: covalent modification, chain-architecture regulation, supramolecular regulation, and food-matrix composite structuring. These modifications and structural regulations alter chain properties, helix association, junction-zone formation, interfacial organization, and phase connectivity, thereby affecting gelation, texture, water retention, emulsion stability, processability, and release behavior. Food applications are evaluated through their structure–function relationships across diverse food systems. Particular attention is given to gastrointestinal disassembly, nutrient digestion, bioactive release, and colonic fermentation. Current evidence strongly supports the role of GG structural regulation in food texture and physical stability, whereas evidence for digestive modulation and physiological effects remains limited and is largely based on formulation-specific in vitro studies. Overall, this review provides a structure-guided framework for application-specific selection of GG intervention strategies.

## 1. Introduction

Gellan gum (GG) is an extracellular anionic polysaccharide produced by *Sphingomonas* species [1,2]. Its linear chain consists of a repeating tetrasaccharide containing two glucose residues, one glucuronic acid residue, and one rhamnose residue [3,4,5,6]. The glucuronic acid units impart negative charge and enable cation-mediated association [1]. During cooling, GG chains form ordered helices, which associate into junction zones and, at a sufficient polymer concentration, interconnect to form a continuous three-dimensional network. Commercial GG is commonly available as high-acyl GG (HAGG) and low-acyl GG (LAGG). HAGG retains acetyl and L-glyceryl substituents and forms soft, elastic gels, whereas LAGG is largely deacylated and forms firmer, more transparent, but more brittle gels [7]. The balance between acyl substitution, molecular weight, and ionic conditions determines the basic texture and stability of GG-containing foods.

These properties have established native GG as a versatile food hydrocolloid [8]. Its main advantages include effectiveness at low concentrations, thermal stability, optical clarity, relatively neutral flavor, and ion-responsive texture control. By adjusting acyl form, polymer concentration, and ionic composition, manufacturers can obtain textures ranging from weak suspending networks to firm gels [1]. Native GG is used in beverages, jellies, desserts, confectionery, dairy and plant-based products, starch-containing foods, and systems requiring particle suspension or shape retention. The two commercial forms also have complementary limitations [9]. LAGG may be brittle and prone to syneresis, whereas HAGG may lack the rigidity required for precise shape retention. GG also has limited primary emulsifying activity, and its hydration and gelation are sensitive to sugars, minerals, pH, heating, cooling, shear, and the order of ingredient addition [10]. These inherent limitations of natural GG likely prevent it from concurrently satisfying processing, storage, sensory, and digestive requirements in complex foods, which strongly justifies the necessity of developing targeted, precise modification approaches to unlock its full application potential.

For clarity, this review distinguishes four structurally distinct levels of GG modification and structural regulation: (1) covalent modification alters the chemical structure of the GG chain through the removal, transformation, or introduction of functional groups or covalent linkages; (2) chain-architecture regulation changes molecular-weight distribution through controlled cleavage of the polysaccharide backbone; (3) supramolecular regulation reorganizes helices, junction zones, gel particles, or networks through ions, thermal history, shear, pH, or solvent conditions without altering the covalent GG backbone; and (4) food-matrix composite structuring refers to non-covalent interactions of GG with proteins, starches, lipids, particles, or other polysaccharides. In this review, the first two categories are defined by the term “modified GG”, whereas the latter two are described as structurally regulated GG. These categories are evaluated separately because they differ in material identity, mechanism, evidence requirements, food applicability, safety assessment, and potential regulatory burden [1,11,12,13].

The functional consequences of GG modification and structural regulation are highly application- and stage-dependent, because changes at one structural level may improve a specific attribute while altering other performance outcomes through intrinsic structure–function trade-offs [14,15]. Diminishing molecular weight may decrease process viscosity but compromise network connectivity [12,16]; enhanced ion-mediated interactions can bolster shape retention while heightening brittleness [1,17,18]; and a stable protein–GG interfacial layer can augment emulsion stability while modifying in vitro digestive breakdown and bioaccessibility [19]. Assessment must consequently encompass more than singular metrics like gel strength, viscosity, or encapsulation efficacy, taking into account the particular food matrix, processing sequence, sensory outcomes, release dynamics, evidence quality, safety, and scalability.

Previous reviews have focused on GG gelation, mixed-polysaccharide systems, manufacturing, functionalized hydrogels, films and coatings, and food applications [11,13,20,21]. These studies have contributed significantly to the understanding of GG structure and functionality [11]. However, most of the investigations have been focused on a certain feature of GG chemistry, assembly behavior or application performance. An overall approach integrating the different levels of GG structural regulation, from polymer modification and chain architecture to supramolecular organization and food-matrix interactions with functional outcomes, digestive behavior, safety considerations and industrial translation is still not developed enough [20,21,22].

**Figure 1:**
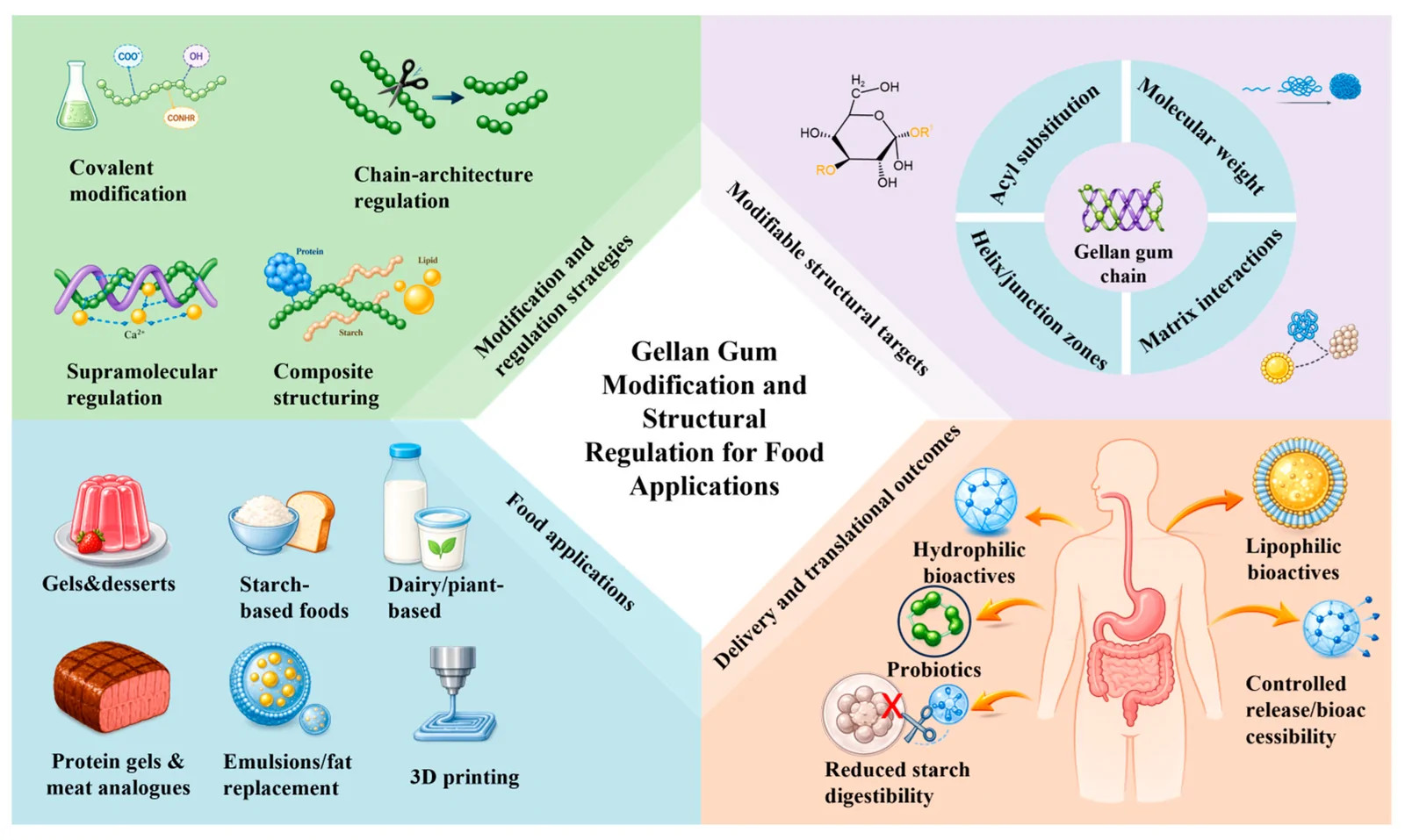
Structure-guided applications and translation of modified and structurally regulated GG.

Accordingly, this review establishes a structure-based framework that classifies GG modification and structural regulation into four categories: covalent modification, chain-architecture regulation, supramolecular regulation, and food-matrix composite structuring (Figure 1). It examines how structural changes at different levels influence processing behavior, rheology, texture, interfacial stability, encapsulation performance, and food functionality, while distinguishing evidence derived from material characterization, model-food systems, simulated in vitro digestion or fermentation, and human studies. Safety and regulatory aspects are discussed as an assessment framework based on material identity, composition, and data requirements rather than as a prediction of regulatory outcomes. This framework aims to facilitate mechanism-guided and application-oriented design of GG systems by linking structural regulation with functional performance, digestive behavior, and translational considerations.

## 2. Structural Basis of Native GG and Targets for Modification

The repeat-unit chemistry, acyl substitution, chain length, and ion-responsive assembly of native GG delimit the structures accessible through modification. Selection of a modification route should begin with the molecular or supramolecular feature that limits performance in the intended food.

### 2.1. Molecular Structure and Assembly of Native GG

**Figure 2:**
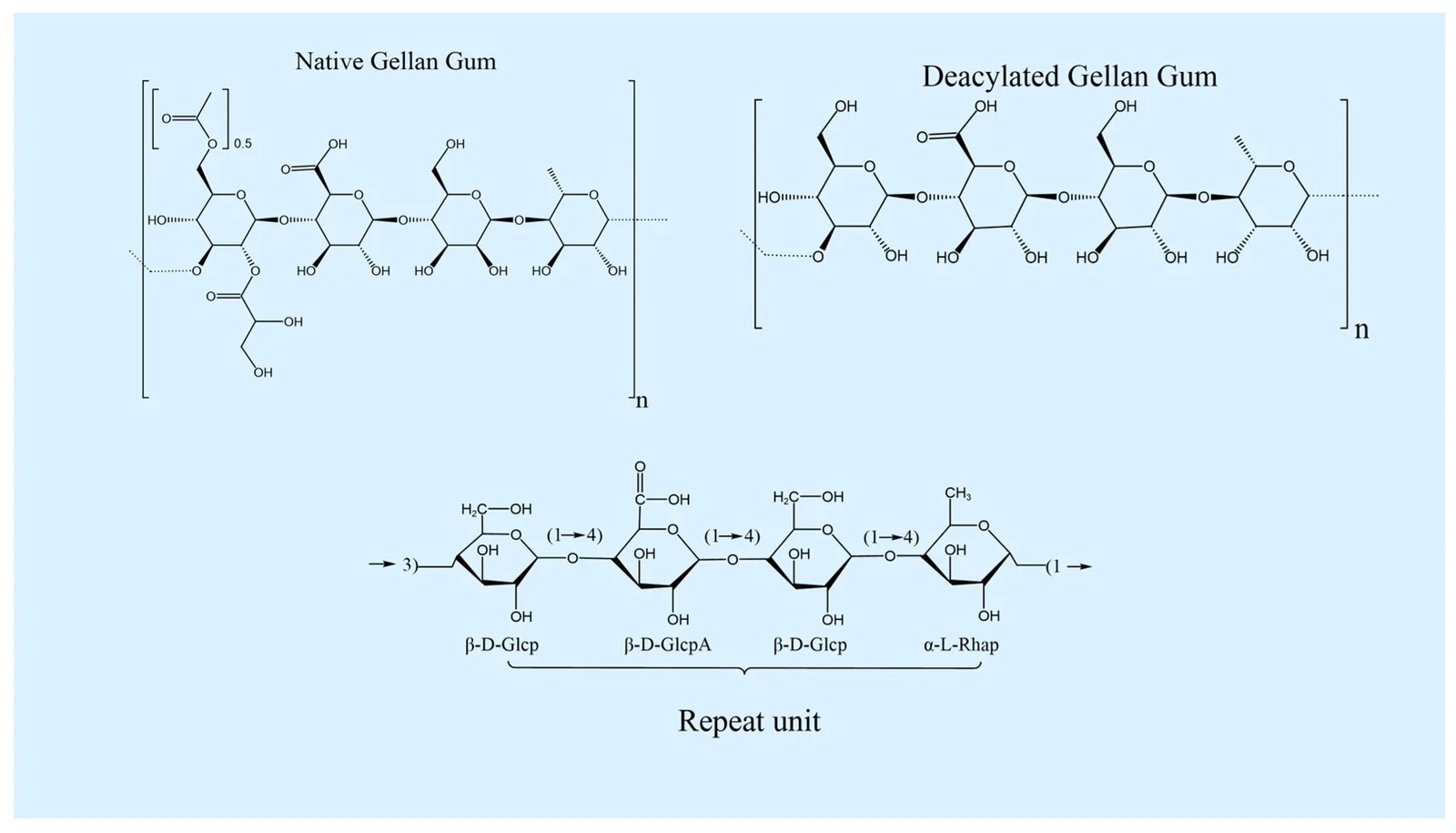
Repeating tetrasaccharide unit of GG.

GG is a linear heteropolysaccharide composed of repeating tetrasaccharide units containing two D-glucose residues, one D-glucuronic acid residue, and one L-rhamnose residue (Figure 2) [3]. This unbranched repeat places hydroxyl groups along the chain and one glucuronic acid carboxylate in each unit [3]. The carboxylates confer anionic character and mediate ion-dependent association [23,24], and the hydroxyl groups support hydration and provide sites for chemical derivatization [25].

Native high-acyl GG carries an L-glyceryl substituent at O-2 of the 3-linked glucose residue and an acetyl group at O-6 of the same residue in part of the repeat units [26]. These substituents impede the tight proximity of neighboring chains and promote hydrated, malleable networks [23,27]. Alkaline processing eliminates them while preserving the tetrasaccharide framework, resulting in low-acyl GG [1,7]. The acyl content serves as a direct method for modifying helix arrangement, flexibility, and fracture characteristics [23,27].

The backbone presents three experimentally unique targets: hydroxyl groups may undergo oxidation or derivatization [25], native acyl substituents can be eliminated [1], and glycosidic linkages can be severed to regulate molecular weight [12]. Glucuronic acid carboxylates bind cations and hence modulate supramolecular organization [1,23,24]. The covalent bonding of a protein imparts interfacial capabilities that GG alone does not possess [28]. These objectives allow for the independent consideration of modifications in covalent chemistry, chain structure, or network formation.

In addition to the main structure, GG chains have a systematic shift from coil to helix during cooling [1,24]. The helices then aggregate via hydrogen bonding and cation-mediated charge shielding to create junction zones and more extensive networks [1,23]. The acyl composition dictates the density of helix packing [23,27], molecular weight determines the bridging of chains across many junctions [12,16], and ionic circumstances influence the degree and consistency of aggregation [1,24]. This hierarchical framework elucidates the exceptional adaptability of GG: modifications at the functional-group, chain, helix, or network levels can yield specific alterations in food performance without necessitating an entirely new polysaccharide backbone.

### 2.2. Structural Characteristics of Native GG in Food Matrices and Structural Regulation Opportunities

Food matrices alter GG assembly through competition for water and coupling among biopolymer phases [11,20]. In multicomponent foods, starch gelatinization [29,30], protein aggregation and interfacial adsorption [15,31,32], lipid-domain organization [16], mixed-polysaccharide interactions [11], and changes in pH [10,33], ionic strength, water activity, and ingredient-addition sequence [34] can all influence GG hydration, helix association, and network formation. Consequently, a treatment that changes isolated GG may produce a smaller, different, or even opposite effect in a complex food matrix. Commercial HAGG or LAGG used without further polymer-level modification is therefore treated as a formulation component rather than evidence of a new modified GG derivative.

The matrix in which GG is incorporated determines the functional requirements and the relevant structural features that need to be regulated [12,15]. From a food-application perspective, these requirements can be summarized into four major aspects: processability (hydration, viscosity, pumpability, and tolerance to heat or shear), macroscopic structural properties (elasticity, fracture behavior, water retention, and shape stability), interfacial and transport functions (suspension stability, emulsion stabilization, encapsulation, and release), and compatibility with oral processing and gastrointestinal disassembly [16,27,29,30]. Importantly, optimizing one functional attribute may not necessarily improve others; therefore, GG regulation should be guided by the specific functional requirement and application constraint rather than by maximizing a single rheological parameter.

To establish the link between structural regulation and functional performance, this review considers four structural determinants of GG behavior: polymer chemistry and chain architecture, helix association and junction-zone formation, gel-particle and interfacial organization, and phase connectivity within complex food matrices. These structural determinants provide the basis for the classification of GG modification and structural regulation strategies in Section 3 and help distinguish direct polymer modification from formulation-driven structural organization.

## 3. Modification and Structural Regulation Strategies of GG

GG modification and structural regulation strategies are classified here according to the primary structural level altered rather than by equipment type or broad categories such as physical, chemical, or enzymatic treatment. Four categories are consistently considered: covalent modification, chain-architecture regulation, supramolecular regulation, and food-matrix composite structuring. Covalent modification and chain-architecture regulation directly alter the GG polymer structure and are therefore defined as modified GG, whereas supramolecular regulation and composite structuring reorganize chemically unchanged GG through non-covalent interactions and are defined as structurally regulated GG systems. A single processing approach may influence more than one structural level; for example, ultrasound can induce chain scission through molecular degradation or facilitate protein–GG conjugation under specific conditions [12,35]. Therefore, classification should be based on direct evidence of the structural changes induced by the treatment rather than the processing method itself.

### 3.1. Covalent Modification

Covalent modification is the permanent alteration of GG caused by the breaking, transformation, or creation of covalent bonds with its natural substituents or polysaccharide backbones. Depending on the type of structural change, these strategies can be divided into four main categories: deacylation, which removes native acetyl and glyceryl substituents [1,7]; oxidation, which converts hydroxyl groups into more reactive carbonyl or carboxyl groups and may be accompanied by chain cleavage [25]; Maillard conjugation, which covalently links GG with proteins through reactions between carbonyl and amino groups [28,36,37]; and other covalent modifications, including esterification, amidation, grafting, and permanent chemical crosslinking [13,38,39]. These approaches differ in reaction mechanism, degree of structural alteration, functional consequences, and suitability for food applications.

#### 3.1.1. Deacylation

**Figure 3:**
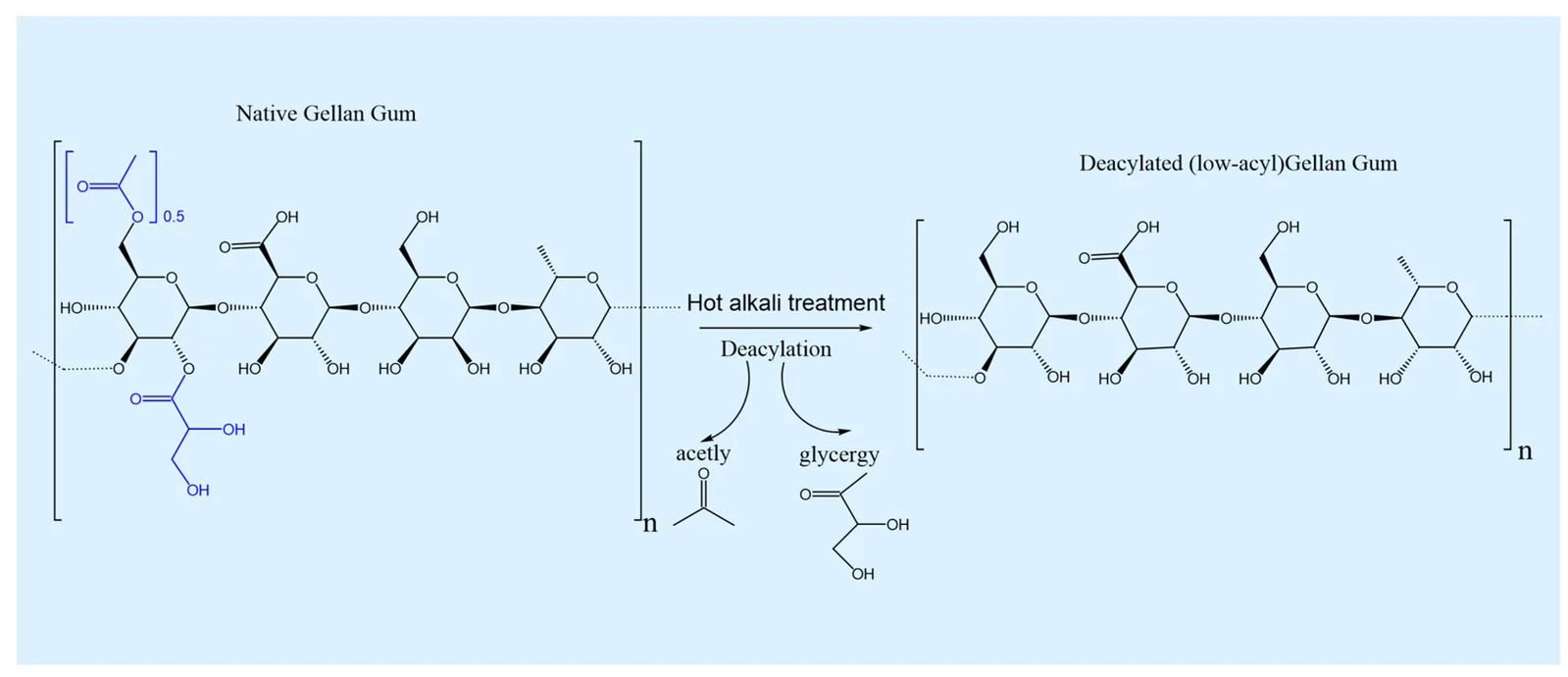
Hot-alkali deacylation of native GG to yield low-acyl GG.

Deacylation is the most established covalent modification, as it is the basis of commercial LAGG synthesis [1,7]. The hot alkaline treatment eliminates most acetyl and glyceryl substituents but the repetitive backbone is substantially retained (Figure 3) [7]. Reduced steric hindrance allows more compact helix association, resulting in increased rigidity and the formation of transparent ion-sensitive gels with high strength but low fracture strain [27,40]. Thus, deacylation is not an unconditional enhancement, but rather a trade-off of elasticity for tight packing. Partial deacylation or blending of HAGG and LAGG can yield intermediate textures [27]. But performance also depends on neutralization, residual salts, molecular-weight loss during processing and subsequent ion setup [16].

#### 3.1.2. Oxidation

**Figure 4:**
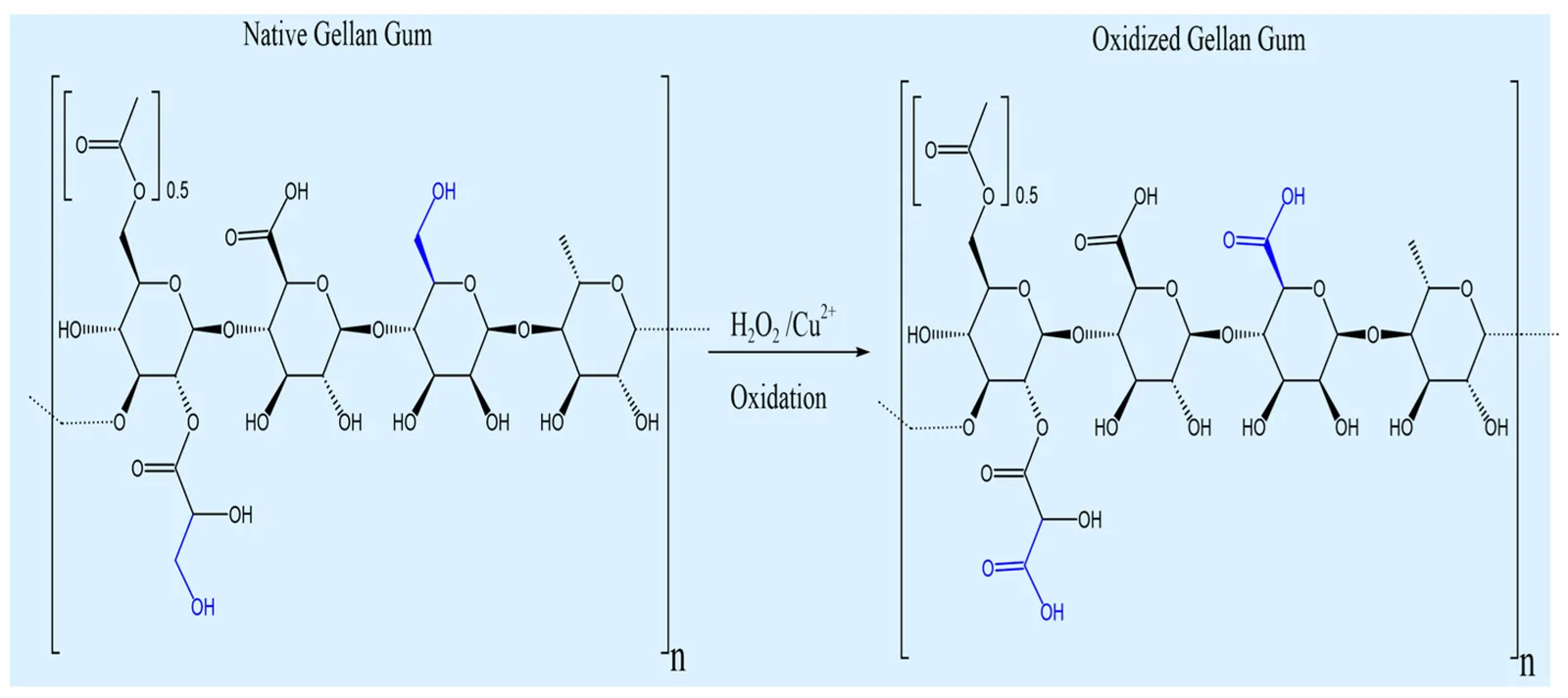
H_2_O_2_/Cu^2+^-mediated oxidation of native GG to yield oxidized GG.

Oxidation can add extra carbonyl or carboxyl groups and may concurrently lead to chain fragmentation. In the H_2_O_2_/Cu^2+^ system documented for GG, an elevated carboxyl content was observed alongside antibacterial coating properties, although there was a reduction in the distinctive ion-induced gelation of the original polymer (Figure 4) [25]. The observed outcome illustrates a compromise between modified surface chemistry and the maintenance of network-forming ability; it does not confirm that oxidation universally enhances GG functioning. In assessing oxidized GG for possible food applications, one must examine the degree of oxidation, alterations in molecular-weight distribution, remaining processing agents, and the generation of low-molecular-weight compounds, as these elements influence the correlation between structural changes, functional efficacy, and safety evaluation.

#### 3.1.3. Maillard Conjugation

**Figure 5:**
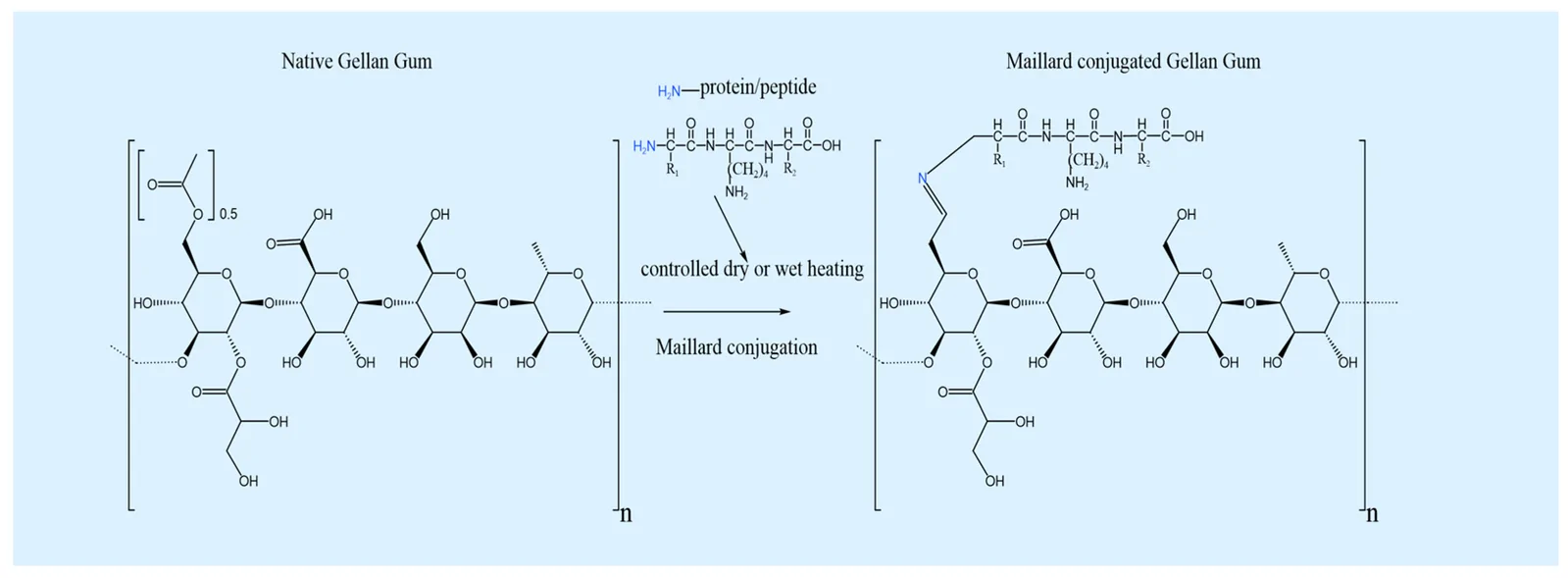
Maillard conjugation of GG with proteins or peptides.

Maillard conjugation forms covalent bonds between GG and proteins through regulated dry or moist heating (Figure 5) [28,35,36,37]. The protein element aids in interfacial adsorption, whereas GG provides charge, steric stabilization, and the structuring of the continuous phase. In soy protein isolate–LAGG systems, covalent bonding has been evidenced by the depletion of free amino groups, an elevated degree of glycation, alterations in spectroscopic properties, and the generation of higher-molecular-weight compounds. Functionality was contingent upon the degree of conjugation: moderate conjugation enhanced certain technological attributes, while high response yielded no reliable supplementary advantage [28].

The outcome of Maillard modification depends strongly on the extent of conjugation, and browning should be regarded only as an indirect indicator rather than definitive evidence of covalent bond formation [28,37]. Insufficient reaction may leave a predominantly non-covalent protein–GG mixture, whereas excessive heating can promote protein aggregation, undesirable color and flavor development, nutrient loss, and the formation of advanced Maillard reaction products [37,41]. Conjugation should therefore be verified by complementary measurements of free amino-group consumption, molecular-weight distribution, spectroscopic changes, browning development, and interfacial functionality, while distinguishing true covalent conjugates from electrostatically associated protein–GG complexes [28,35,36]. For food applications, reaction time, temperature, water activity, and purification conditions should be reported as key processing parameters, while protein allergenicity and Maillard-derived products require separate safety assessment.

#### 3.1.4. Other Covalent Modifications

Esterification, grafting, and permanent chemical crosslinking demonstrate that the GG backbone can support a wider range of functional groups, but most reported systems were designed for biomedical or materials applications rather than foods [13,38,39,42]. In a food field, these routes primarily define the boundary of the field: a chemically feasible GG derivative is not food relevant until its composition, residual reagents, digestive fate, exposure, and regulatory route are established. At present, deacylation, controlled oxidation, and protein conjugation have the most direct food-facing evidence among covalent routes [1,11,36].

### 3.2. Chain-Architecture Regulation

Chain-length regulation differs from functional-group substitution because its primary output is a distribution of shorter polymers or oligosaccharides [43,44]. The technological benefit is lower viscosity [12,16] or faster disassembly, but the corresponding cost is reduced entanglement, fewer network-spanning chains, and even loss of gel formation [44].

#### 3.2.1. Depolymerization

Depolymerization is the cleavage of glycosidic bonds along the GG backbone [12,42,45]. Its immediate structural effects are shorter contour length, lower hydrodynamic volume, and fewer chain entanglements and network-spanning segments [12,16]. These changes are mechanistically different from deacylation, which removes substituents, and from oxidation, which introduces new functional groups and may only secondarily cause chain scission. A valid depolymerization study must therefore demonstrate a shift in molecular-weight distribution and, where chemical treatments are used, determine whether acyl content or backbone chemistry has changed concurrently [42,43].

The functional response is governed by the extent of cleavage [12,46]. Moderate chain shortening lowers solution viscosity and weakens large-scale network connectivity [12,16,47], yet chains may remain long enough to undergo coil-to-helix ordering and participate in ion-mediated junction zones. Ultrasound is a mechanical wave with a frequency above the human audible range (>20 kHz) [48,49,50]. In ultrasonicated LAGG, the pronounced loss of fracture strength relative to the smaller change in thermal ordering supports this separation between retained local association and weakened network connectivity [12,46]. As molecular weight is reduced further, the number of chains able to bridge junction zones falls below that required for a load-bearing network, producing weak gels or non-gelling fractions [44,47]. Depolymerization should thus be treated as a controlled transition from a network-forming polymer to soluble fragments, with molecular-weight distribution, viscosity, gelation, and mechanical properties assessed together.

#### 3.2.2. Low-Molecular-Weight GG

**Figure 6:**
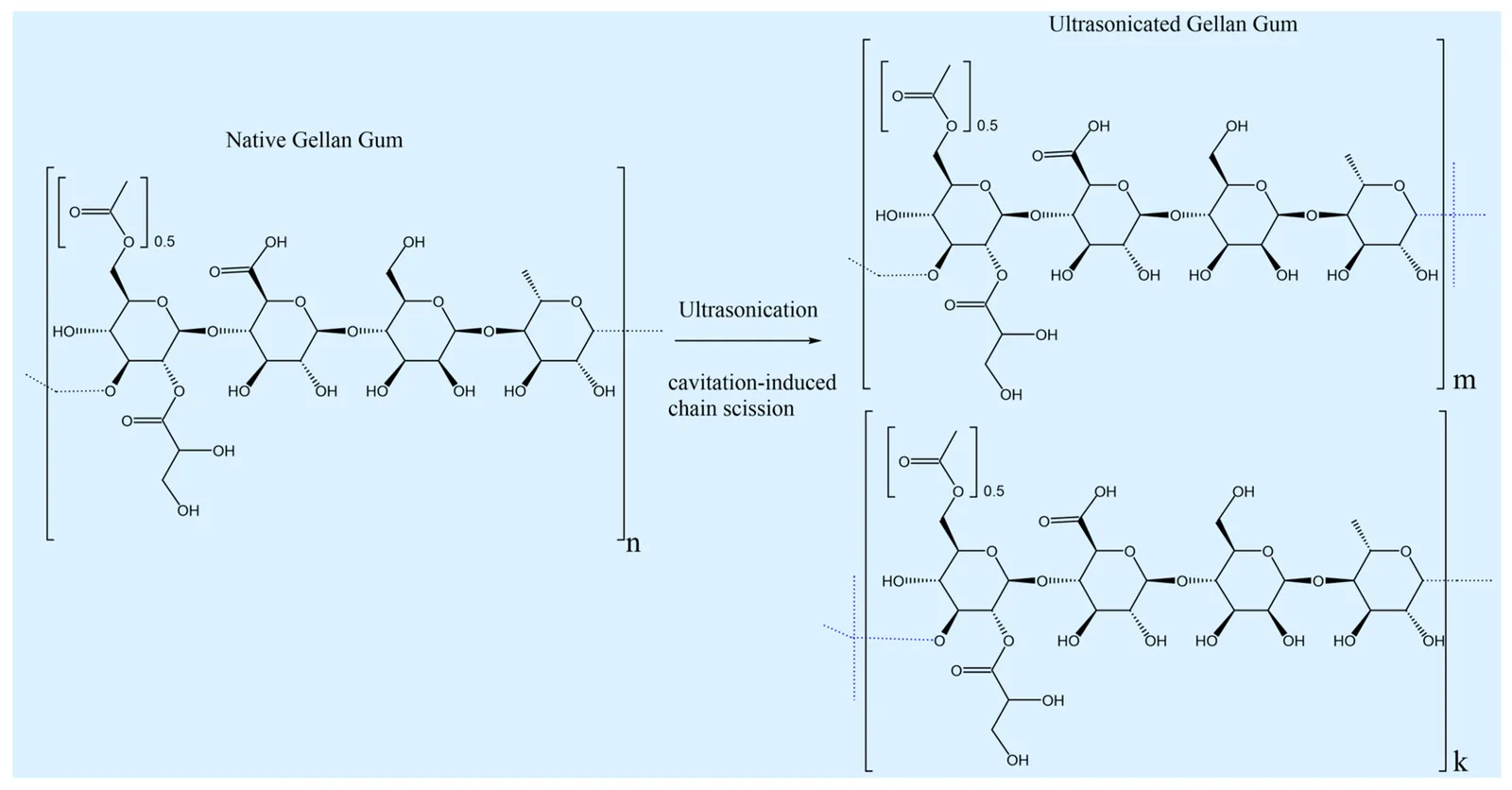
Ultrasound-induced depolymerization of native GG. Noting: m < n, k < n.

Low-molecular-weight GG (LMW-GG) can be generated by physical, chemical, or enzymatic cleavage, but the routes differ in selectivity and product definition. Ultrasound exhibits cavitation effects that can disrupt intermolecular interactions and regulate the structure and functionality of materials [46,51,52]. Ultrasonication can reduce LAGG molar mass and weaken large-deformation gel strength while retaining part of its thermoreversible ordering after moderate treatment (Figure 6) [12]. Irradiation-assisted preparation provides a different route: gamma irradiation first shortens GG chains and is followed by dilute-acid hydrolysis when oligosaccharide production is intended [43]. Because dose, acoustic energy density, temperature, and reaction environment influence product distribution, a fall in viscosity alone should not be used as proof of a defined chain-architecture change [12,43,47].

Chemical hydrolysis can also regulate GG molecular weight. Alkaline hydrolysis has been used to prepare LMW-GG products, but it may affect molecular-weight distribution and acyl substitution simultaneously [47]. In irradiation-assisted production of GG oligosaccharides, dilute-acid hydrolysis is applied after gamma irradiation to further cleave shortened GG chains [43]. Accordingly, molecular-weight distribution and acyl content should be characterized together when assigning the structural consequence of chemical cleavage [43,47].

**Figure 7:**
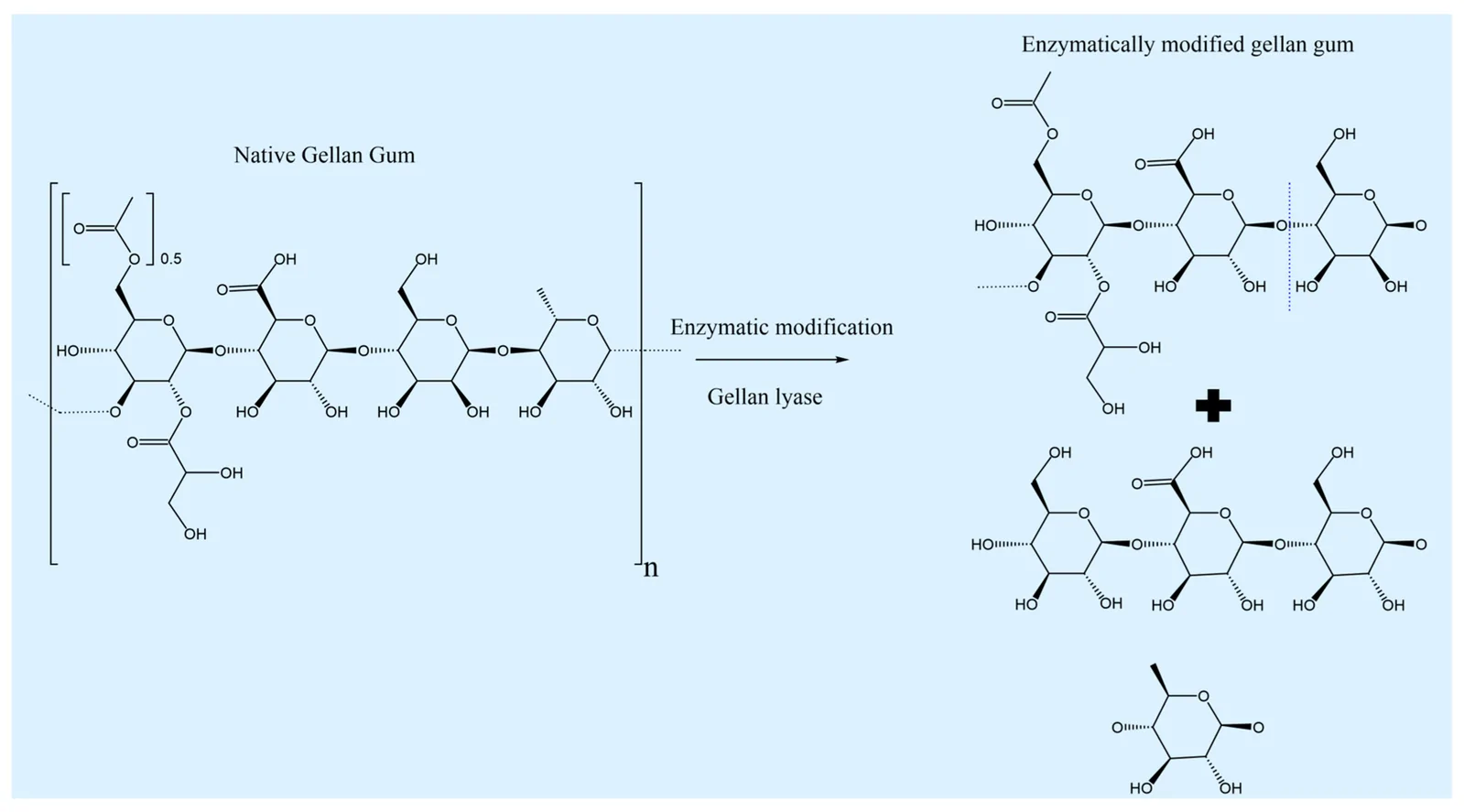
Gellan lyase-mediated enzymatic depolymerization of native GG.

Enzymatic depolymerization provides a linkage-selective route for GG cleavage under relatively mild conditions (Figure 7) [53,54]. Gellan lyases from *Bacillus* sp. GL1 and other gellan-degrading microorganisms can cleave defined linkages in the deacylated GG backbone and generate structurally characterized unsaturated products [45,55,56,57]. Product distribution nevertheless depends on enzyme specificity, substrate acylation and aggregation, reaction time, and downstream separation. Enzymatically produced LMW-GG should therefore be defined by molecular-weight distribution and product composition rather than by reduced viscosity alone.

#### 3.2.3. Oligosaccharides

GG oligosaccharides are discrete short-chain products and should not be treated as interchangeable with polymeric LMW-GG. Their identity requires a degree-of-polymerization profile and structural confirmation of the constituent saccharides and chain ends. Irradiation followed by dilute-acid hydrolysis produced GG oligosaccharides dominated by degrees of polymerization of four and eight, consistent with cleavage around the tetrasaccharide repeat [42]. Gellan lyases can generate more linkage-defined products under mild conditions [45,53], whereas broader sphingan lyases may also act on related sphingan polysaccharides [58]. Evidence obtained with non-GG sphingans cannot be transferred to GG oligosaccharides without product-level characterization.

The potential food value of GG-derived oligosaccharides is related to their possible biological functions beyond the gel-forming properties of native GG. Irradiation–acid hydrolysis products have been investigated as GG oligosaccharide fractions under in vitro conditions [43,59], while related sphingan oligosaccharides provide supportive but non-equivalent evidence [60]. These studies suggest a potential microbial use of GG-derived oligosaccharides in controlled animals, but do not show a verified prebiotic impact in people. Further validation will involve characterization of product composition, gastrointestinal stability, microbial selectivity, effective dose, tolerance and reproducibility in relevant biological models and human research.

### 3.3. Supramolecular Regulation

The supramolecular structure of GG is formed through the non-covalent association of chemically unchanged polysaccharide chains. During cooling, disordered GG chains first adopt ordered double helices. These helices subsequently aggregate into junction zones through hydrogen bonding and cation-mediated interactions. Interconnection of the junction zones produces either a continuous gel network or, under shear, dispersed gel particles [1,24,61].

Acyl substitution, molecular mass, ion identity and concentration, temperature, shear, pH, and co-solutes influence GG assembly from coil–helix ordering through helix aggregation and network formation [1,17,62,63,64]. These variables regulate supramolecular organization without necessarily changing covalent polymer identity. Accordingly, evidence for a change in rheology or gel texture should be attributed to the measured level of assembly rather than described generically as polymer modification.

#### 3.3.1. Ion-Mediated Regulation

Cations regulate GG by reducing electrostatic repulsion between glucuronic acid residues and promoting helix aggregation. Divalent ions generally create efficient junctions, whereas monovalent ions mainly screen charge; excessive or poorly distributed ions can cause rapid local aggregation, heterogeneity, and syneresis [17,18,24]. Ion identity, activity, addition sequence, and water mineral content must be treated as process variables [65]. Ion-mediated regulation is powerful precisely because it is reversible at the formulation level, but it does not create a new covalent GG derivative.

#### 3.3.2. Thermal Regulation

Temperature predominantly regulates conformational and network changes in GG. Heating increases hydration and breaks pre-existing helices or aggregates, whereas cooling promotes ordered helix formation and subsequent aggregation [1]. Even with a given composition, thermal treatment can affect elasticity and network homogeneity [62]. These effects are considered supramolecular regulation rather than chemical change.

#### 3.3.3. Shear-Induced Fluid-Gel Formation

**Figure 8:**
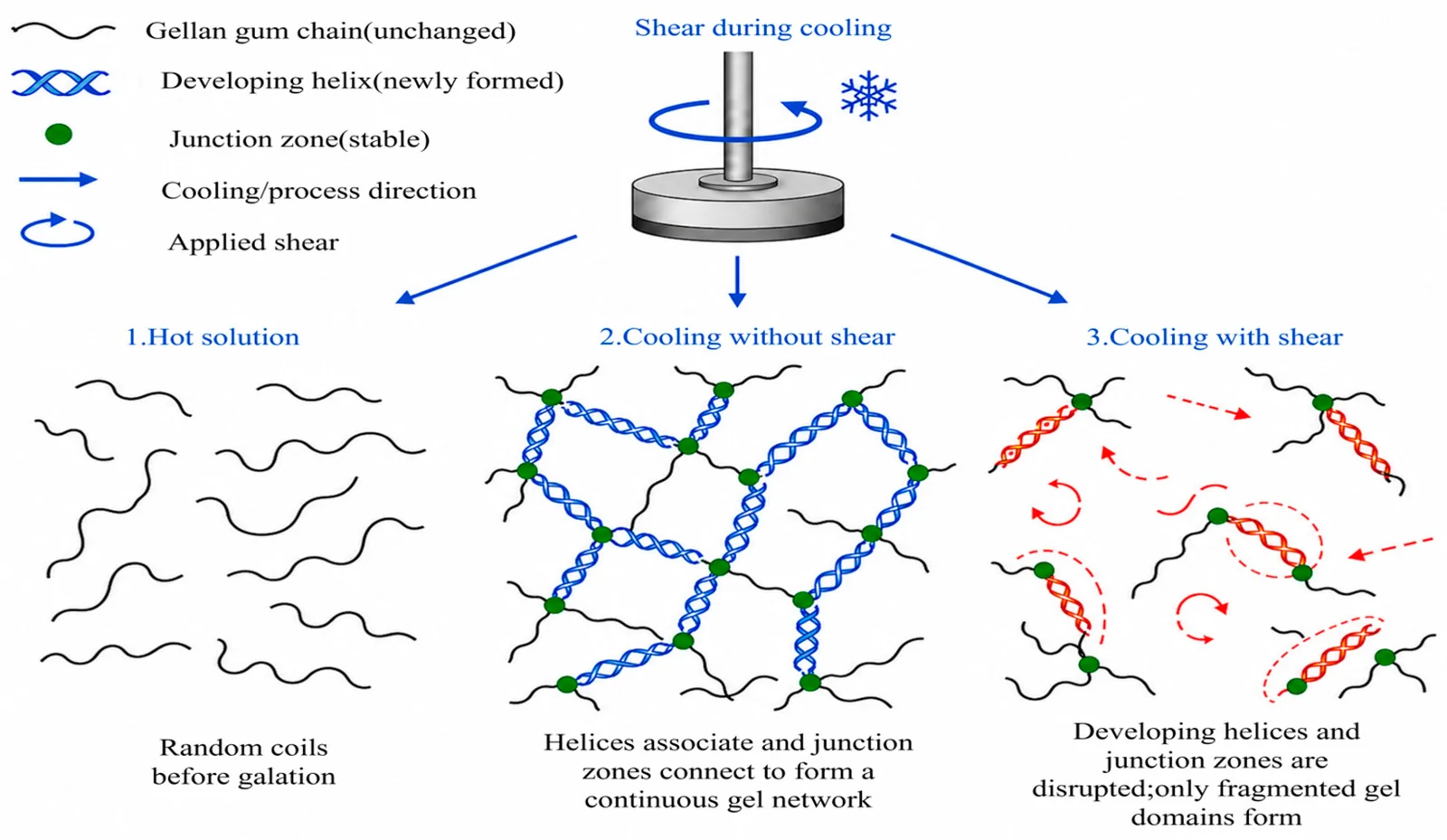
Effect of shear during cooling on GG gelation.

Shear applied during cooling reorganizes the GG supramolecular structure (Figure 8). It disrupts the construction of a continuous network, resulting in gelled microdomains dispersed across a continuous phase, often known as a fluid gel [61,64,66]. The stirring rate, temperature profile, cation type, and GG content all influence particle morphology, yield behavior, low-shear viscosity, and physical aging [61,64,66,67]. Instead of a chemically new GG chain, the gel particle serves as the functional unit.

#### 3.3.4. pH, Sugars, and Solvent Environment

pH and co-solutes control the equilibrium of hydration and association. Acidification alters GG charge screening and can cause structure in acid-sensitive fluid gels, whereas sugars lower water activity [68], change solvent quality, and affect both gel mechanics and taste transmission [63,65,69]. These effects are significantly formulation-dependent since proteins and minerals react to the same variables. pH or sugar adjustment should be presented as environmental control of GG assembly, rather than proof of polymer alteration.

### 3.4. Food-Matrix Composite Structuring with GG

Food-matrix composite structuring involves combining GG with proteins, carbohydrates, lipids, particles, or other polysaccharides while keeping the covalent GG backbone. Electrostatic interactions, hydrogen bonding, ion bridging, water partitioning, excluded volume effects, phase separation, and interpenetration of independently generated networks all contribute to the structure [11,31,32]. Because these systems are formulation-level structures rather than novel GG derivatives, they are treated separately from direct polymer alteration throughout this review.

#### 3.4.1. GG–Proteins

Protein–GG composites demonstrate how compatibility is more important than the strength of either component alone. GG can influence protein aggregation, create a secondary ion-set network, and redistribute water [70]. In thermally induced pea-protein gels, GG increased water retention and formed a denser microstructure, but the results varied depending on concentration and gelation circumstances. In potato-protein systems, HAGG favored a more homogenous composite network, but LAGG-rich formulations increased stiffness and brittleness, potentially promoting spatial separation of protein- and polysaccharide-rich domains [31,71]. A stronger LAGG gel does not necessarily result in a stronger or more acceptable protein composite.

Process sequencing is very significant in plant-based protein meals. Calcium can filter protein charge, causing aggregation while also cross-linking GG [72]. If GG hydrates too soon, it can sequester water or increase viscosity before the proteins spread and align. In meat-analog formulations, delayed inclusion of LAGG following protein hydrolysis increased mechanical strength and anisotropy more than premature incorporation [73]. These findings demonstrate that composite modification entails controlling hydration, acidification, ion addition, shear, and chilling, rather than only ingredient ratio.

#### 3.4.2. GG–Starches

GG–starch composites are influenced by water competition, the relative timing of starch gelatinization and GG assembly, and the continuity of the two phases [11,30,74,75]. GG can alter starch pasting and matrix cohesion, but the magnitude and direction depend on acyl form, concentration, ionic conditions, and processing history [30,75]. Based on these findings, viscosity changes should be interpreted together with microstructural, water-mobility, and starch-accessibility measurements to distinguish network reinforcement from physical restriction or phase separation [11,74].

#### 3.4.3. GG–Lipids/Emulsions

In emulsions, HAGG can stabilize dispersed droplets primarily through continuous-phase structuring and reduction in creaming, whereas sodium caseinate–HAGG systems additionally involve protein adsorption and polysaccharide-associated interfacial stabilization [32,76,77]. GG-containing networks can also alter simulated digestive transformations and bioactive release [14,19,78]. These observations support matrix- and interface-level regulation; they do not imply that conventional HAGG emulsions are covalently modified GG systems.

Based on the emulsion studies summarized above, effective composite design should consider three coupled structures: the adsorbed interfacial layer, the GG-containing continuous phase, and the coupling of droplets to the surrounding gel network [14,32,76,77]. From this standpoint, studying GG purely as a viscosity modulator may ignore interfacial and digestive effects. This statement is a synthesis of the evidence presented rather than a mechanism proved in a single study.

#### 3.4.4. GG–Polysaccharides

**Figure 9:**
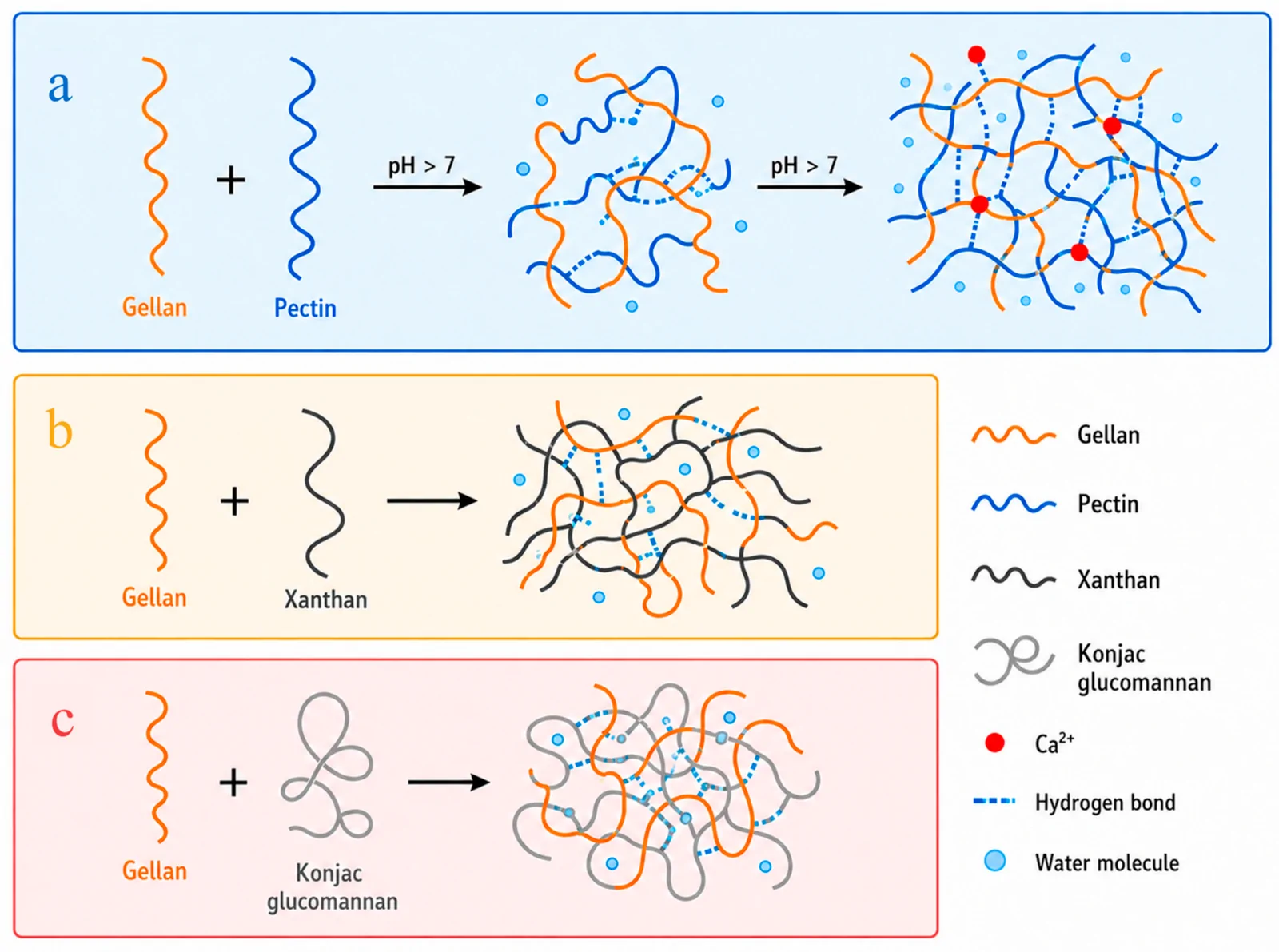
Schematic representation of composite networks formed by gellan gum with different polysaccharides: (**a**) gellan–pectin; (**b**) gellan–xanthan; and (**c**) gellan–konjac glucomannan. Dashed lines indicate hydrogen bonds, blue circles indicate water molecules, and red circles indicate Ca^2+^ ions.

Mixed-polysaccharide GG systems can produce synergistic, interpenetrating, or phase-separated structures depending on composition and processing circumstances [11,79,80]. Curdlan–GG gels exhibit composition-dependent synergistic behavior [79], while gelatin–GG–inulin systems show changes in water distribution and network elasticity [80]. Increased modulus in GG-based food systems may not always suggest a specific chemical process, since greater viscoelasticity can be caused by changed chain organization, helix aggregation, junction-zone formation, or GG interactions with matrix components. To better understand how GG organization translates into functional performance, rheological data should be combined with additional microstructural, water-distribution, and phase-behavior analyses (Figure 9) [11,79,80].

### 3.5. Comparison of GG Modification and Structural Regulation Strategies

Based on the findings presented in Section 3.1, Section 3.2, Section 3.3 and Section 3.4, GG modification and structural regulation approaches are divided into four categories: covalent modification, chain-architecture regulation, supramolecular regulation, and food-matrix composite structure [11,16,35]. The first two categories directly affect the GG polymer, but the last two influence the organization and interactions of chemically unmodified GG [11,16]. Because these techniques act at different structural scales and fulfill distinct functional requirements, their results cannot be measured purely using broad phrases like “improved functionality.” Alterations in viscosity, modulus, encapsulation efficiency, or storage stability are only meaningful when linked to validated structural alterations and specific food-application goals [11,16,22].

For GG-based food systems, route selection should start with determining the specific structural limitation that limits the desired application performance [11,16,35]. When the fundamental chemical functionality of GG needs to be changed, covalent modification can introduce new functional groups or interfacial contacts, but it must be verified for chemical alterations, molecular integrity, and residual reagents or reaction products [19,25,36]. When gel behavior is restricted by molecule size or chain organization, chain-architecture modulation can be utilized to modulate molecular-weight distribution and network-forming capabilities, but changes in gel strength and processability must be carefully assessed. Supramolecular regulation via acyl form, ionic environment, heat history, or shear processing allows for the tuning of network structure without changing the polymer backbone in applications dictated by GG helix association, junction-zone creation, or ion-mediated aggregation [11,16,22,35]. In composite food systems, GG functionality is determined by its interactions with proteins, starches, lipids, or other polysaccharides, necessitating the study of phase organization and non-covalent interactions [11,16,31,81]. Therefore, the most appropriate GG regulation strategy is not determined by the extent of structural alteration, but by whether the selected approach effectively addresses the application-specific structural requirement while maintaining functional performance and practical feasibility.

No single GG regulation strategy is universally superior because each approach modifies a different structural feature and addresses different functional objectives [11,16,35]. Directly modified GG, supramolecularly regulated GG networks, and GG-containing composite systems should therefore be evaluated within their corresponding structural category and evidence level [11,22,35]. A rational GG design strategy requires linking molecular changes, helix and network organization, food-scale functionality, digestive behavior, safety considerations, and translational feasibility rather than relying on generalized claims of improved performance [22,35]. Table 1 summarizes these strategies according to structural output, food functionality, translational burden, and validation level.

## 4. Food Applications of Modified and Structurally Regulated GG

**Figure 10:**
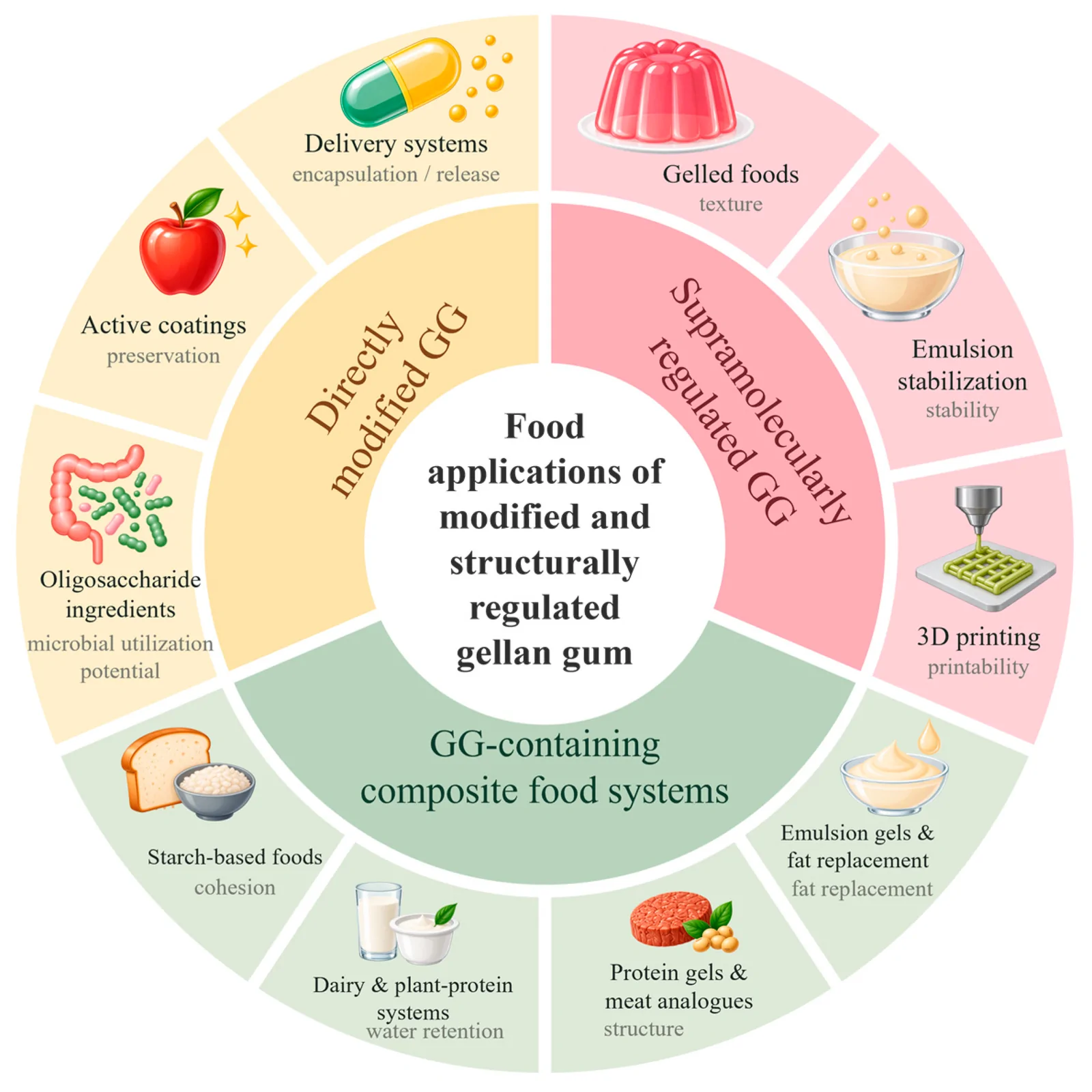
Representative food applications of modified and regulated GG.

To align material identity with application evidence, this section separates directly modified GG from supramolecularly regulated GG and GG-containing composite food systems (Figure 10). Direct applications of covalent or chain-architecture-modified GG are discussed first; conventional HAGG/LAGG formulations structured by ions, heat, or shear are then treated as supramolecular regulation; and non-covalent mixtures with food proteins, starches, lipids, or other polysaccharides are treated as composite food systems. Table 2 summarizes representative formulation conditions, quantitative outcomes, and validation levels.

### 4.1. Applications of Modified GG

Directly modified GG currently has a narrower food-application evidence base than conventional GG structuring [25]. The clearest food-facing examples are protein–GG Maillard conjugates used in emulsion or fat-replacement systems and oxidized GG evaluated in active coatings. Chain-architecture-modified GG is supported mainly by material characterization and functional-property evaluation, whereas GG-derived oligosaccharides have been investigated additionally through microbial utilization or in vitro fermentation models rather than validated final-food applications [28,35,36,43].

#### 4.1.1. Protein–GG Maillard Conjugates

Covalent protein–GG conjugates provide interfacial and gelation functions that differ from non-covalent protein–GG mixtures. In cold-set aerated SPI–GG conjugate gels developed as mayonnaise fat replacers, optimized conditions included 1.5% SPI, 300 mM CaCl_2_, and 90 min heating, with an approximately 35% glycation degree and a 20.8% higher overrun than the corresponding non-conjugated system [84]. Formulations with up to 50% oil replacement retained favorable sensory acceptance under the reported conditions [84]. WPI–LAGG conjugates have also been used to stabilize beta-carotene emulsions, improving resistance to pH, salt, heating, freezing, and storage while altering in vitro digestive fate [19,36]. These applications represent direct covalent modification and should not be merged with ordinary protein–GG composites.

#### 4.1.2. Oxidized GG in Active Coatings

Hydrogen-peroxide/Cu^2+^-oxidized GG provides a direct example of chemically modified GG applied to food surfaces. The reported derivative showed increased carboxyl functionality and antimicrobial coating performance, but it also lost the characteristic ion-induced gelation of native GG [25,96,97]. The application evidence therefore supports a coating-specific trade-off rather than a general improvement in GG functionality. Translation requires characterization of oxidation degree, chain scission, residual oxidant/catalyst, migration, and exposure in addition to preservation performance [25,83].

#### 4.1.3. Chain-Architecture-Modified GG: Limited Food Validation

Ultrasound-, chemical-, enzymatic-, and irradiation-assisted cleavage can generate LMW-GG or GG oligosaccharides [12,43,45,47]. However, most available studies emphasize molecular-weight distribution, rheological behavior, gelation properties, product composition, or model-system evaluations rather than performance in finished food systems [12,43,47,59]. These materials should therefore be described as emerging food-relevant fractions rather than established application platforms. Their use is most plausible where lower process viscosity, faster disassembly, or fermentability is required, but direct final-food and human validation remains limited.

This evidence gap is important because a lower molecular weight does not by itself predict improved processing, digestion, or physiological function. Product definition should include molecular-weight or degree-of-polymerization distribution, acyl content, chain-end composition, residual processing agents, and retained gelation before application claims are compared across studies [16,43,47].

### 4.2. Applications of Supramolecularly Regulated GG

#### 4.2.1. Gelled Foods

Gelled foods commonly use mixed HAGG/LAGG systems and ion- or thermally regulated GG networks rather than new GG derivatives. Mao et al. evaluated 0.5–1.5% total GG at HAGG/LAGG ratios of 25/75, 50/50, and 75/25 with 2–80 mM Ca^2+^ and reported failure strains of approximately 0.6–1.5, with the strongest synergistic response near a 50/50 HAGG/LAGG ratio [4]. Thus, acyl ratio and ion level provide quantitative formulation variables for balancing firmness and deformability rather than evidence of a chemically modified polymer.

At a fixed polymer content, changing the HAGG/LAGG ratio and cation level can shift the same formulation from a weak elastic structure toward a stronger but less deformable network [4,17,18,98]. Reduced-sucrose GG gels also show formulation-dependent changes in compression resistance and flavor release [99,100]. These systems should therefore be reported as supramolecularly regulated GG with defined composition and process history.

#### 4.2.2. Emulsion Stabilization

HAGG can stabilize oil-in-water emulsions primarily through continuous-phase structuring. In emulsions containing 30% sunflower oil, 0.01–0.20% HAGG was evaluated and phase-stable emulsions were obtained at HAGG concentrations above 0.05% (*w*/*w*), whereas LAGG did not stabilize emulsions under the same preparation conditions [77]. Sodium caseinate–HAGG systems provide additional interfacial stabilization [32,76,101,102]. These are supramolecular or matrix-level effects because the GG backbone is not covalently modified.

#### 4.2.3. Three-Dimensional Printing Foods

Three-dimensional printing primarily uses thermally or ionically regulated GG networks and GG-containing inks. Different hydrocolloids exhibit distinct properties and printing behaviors in three-dimensional food printing [103,104,105]. In a corn-starch printing system, 4% and 6% GG produced clear solidification/plastic-deformation behavior after extrusion, and the temperature-sensitive GG transition enabled curing in approximately the 30–45 °C range [87]. GG concentration also altered curing rate, hardness, and adhesion [87]. Other GG-containing emulsion gels and bigel inks have been optimized through rheological and print-fidelity measurements [90,91,105,106,107]. These quantitative parameters help clarify the relationship between GG formulation, processing conditions, and printing performance, whereas qualitative descriptions of shape retention alone provide limited insight into the underlying structure–function relationship.

For dysphagia-oriented applications, print fidelity alone is insufficient. Final GG-containing structures should also be evaluated for bolus cohesion, adhesiveness, lubrication, oral residue, nutritional density, sensory acceptance, and relevant IDDSI criteria [90,91,92,106]. Current evidence therefore supports printability and texture regulation more strongly than clinical swallowing benefit.

### 4.3. GG-Containing Composite Food Systems

#### 4.3.1. Starch-Based Foods

Starch-based foods use GG–starch composite structures formed during coordinated starch gelatinization, cooling, and GG assembly. In jasmine rice, 1–3% LAGG increased hardness by approximately 8–26% and chewiness by about 1.4–2.3-fold, whereas effects in parboiled rice were smaller [29]. The same study reported an approximately 10–22% reduction in predicted glycemic index [29]. Earlier work also showed reduced in vitro starch hydrolysis in GG-structured white rice under defined processing conditions [74].

A randomized crossover study subsequently showed a lower acute postprandial glycemic response for one defined LAGG-structured rice formulation [93]. This human finding supports a formulation-specific effect and should not be generalized to all GG-containing starch foods. These applications are best described as food-matrix structuring because rice cooking with LAGG does not introduce a new covalent modification of the GG chain.

#### 4.3.2. Dairy and Plant-Protein Systems

Dairy and plant-protein systems generally involve GG–protein composite structuring. In thermally induced pea-protein gels, lower GG concentrations (≤0.1%) showed limited gel-network formation, whereas 0.4% GG promoted the development of a relatively dense and stable structure [31]. In egg-based yogurt, 0.045% GG gave the highest reported sensory score (83.57), while 0.085% GG increased water-holding capacity by 30.35%, hardness from 10.98 to 13.76 g, and adhesiveness from 8.25 to 14.95 g·s relative to the control [89]. These data illustrate concentration-dependent matrix regulation and also show that excessive GG can create undesirable hardness or stickiness.

#### 4.3.3. Protein Gels and Meat Analogs

In plant-protein meat analogs containing pea-protein isolate and wheat gluten, 2% LAGG was selected after screening 0–3% GG because increasing GG strengthened the product [73]. GG-free samples showed anisotropy indices at or below 1, whereas GG-containing systems exhibited indices above 1; the most favorable fibrous structure was obtained when LAGG was added after pea-protein hydration in the presence of CaCl_2_ [73]. The result demonstrates that processing sequence and matrix assembly, rather than polymer identity alone, determine application performance.

#### 4.3.4. Emulsion Gels and Fat-Replacement Systems

In HAGG-based emulsion gels, the continuous polysaccharide phase significantly contributes to viscoelastic behavior, whereas droplets provide limited reinforcement and can impair creep compliance [108]. Composite fat-replacement systems should therefore report GG concentration, oil fraction, rheological parameters, and droplet–matrix interactions. When covalent SPI–GG conjugates are used instead, as in the aerated-gel mayonnaise system discussed in Section 4.1.1, the material should be classified separately because its chemistry and translational requirements differ [84].

#### 4.3.5. Composite Edible Coatings

Non-covalent GG composite coatings combine the GG film-forming phase with other polysaccharides or active compounds. In one blueberry system, an optimized coating contained 0.22% thymol microcapsules and 0.075% total polysaccharides at a KGM:LAGG ratio of 1:2; storage at 2 ± 0.5 °C extended shelf life to 42 d, approximately three times that of the control [60]. Gellan-based coatings containing oregano essential oil also reduced weight loss in mandarins under the reported cold-storage conditions [109]. These outcomes are commodity- and formulation-specific and should not be treated as evidence for a chemically modified GG derivative.

## 5. GG-Based Delivery Systems and Digestive Behavior: Evidence and Limitations

**Figure 11:**
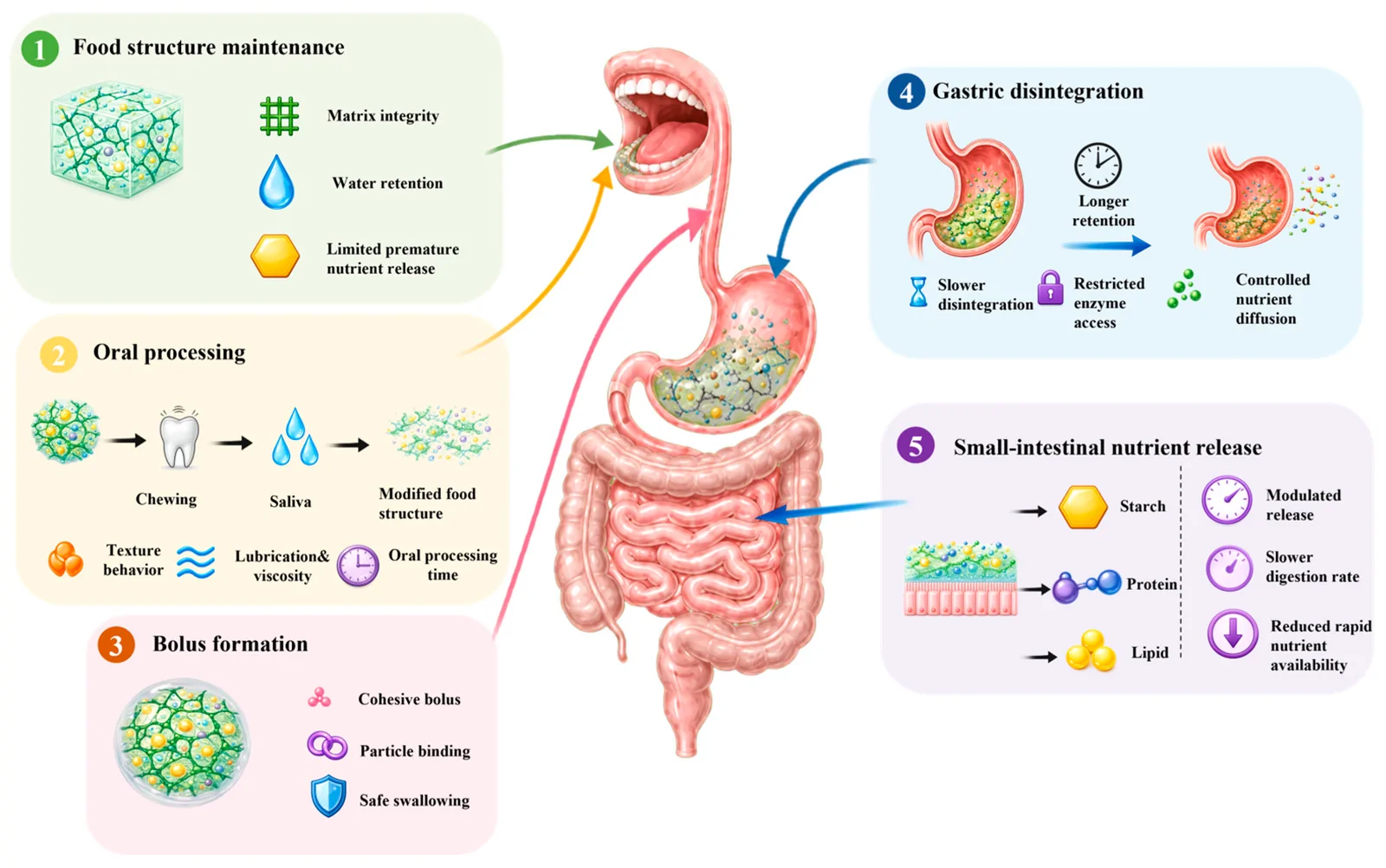
Schematic illustration of the potential roles of GG-structured food matrices during oral processing and gastrointestinal digestion.

GG-containing delivery systems can alter gastrointestinal disassembly through changes in chain, network, particle, or interfacial organization (Figure 11). Acyl content and molecular weight affect chain flexibility and junction-zone connectivity, while ions, heat, and shear influence crosslink density and whether GG forms a continuous gel, gel particles, or a weakly structured solution [12,24,61]. However, the evidence supporting digestive effects is substantially less extensive than that supporting rheology and food structuring. Most studies reviewed here use formulation-specific in vitro digestion or fermentation models. Direct human evidence is limited to the randomized crossover study of LAGG-structured rice [93], and comparable animal validation is not available for most of the modified or structurally regulated systems summarized below. Accordingly, this section distinguishes observed in vitro outcomes from experimentally demonstrated mechanisms and from proposed physiological implications.

### 5.1. Structural Principles and Evidence Scope

Different GG modification and structural regulation strategies influence delivery behavior through distinct structural pathways, including junction-zone formation, chain mobility, cargo–matrix interactions, interfacial persistence, particle architecture, and coating continuity. Deacylated LAGG is appropriate for ionically structured carriers due to its ordered helices that produce compact cation-mediated junction zones. In simulated gastrointestinal digesting investigations, gelatin–GG hydrogels, double-emulsion-filled GG hydrogels, and chitosan-coated GG-containing particles showed formulation-dependent gastric protection and intestinal release behavior [110,111,112]. Another approach is molecular-weight modulation, which alters chain connectivity and transport resistance: moderate depolymerization improves processability and matrix accessibility, but substantial cleavage diminishes GG chains’ ability to form continuous diffusion barriers [12,16,44]. Protein–GG Maillard conjugates and GG-containing composite carriers further influence delivery via interfacial stability, electrostatic interactions, and multicomponent network creation. WPI-LAGG conjugate-stabilized emulsions, for example, demonstrated lower beta-carotene bioaccessibility during simulated gastrointestinal digestion, which was linked to altered interfacial and rheological properties [19,36]. However, the relative contributions of interfacial resistance, protein hydrolysis, lipid digestion, and micelle formation require additional investigation.

Current evidence for GG-based delivery systems is primarily based on material characterization and simulated gastrointestinal models, with limited direct validation of in-testinal absorption, bioavailability, and physiological effects [110,111]. As a result, encapsulation efficiency, storage stability, stomach retention, or higher in vitro release should not be taken as independent indicators of improved delivery performance. Instead, carrier examination should consider structural stability, gastrointestinal deconstruction, digestive accessibility, and, if available, absorption or physiological effects [112,113]. In this review, bioaccessibility refers to the proportion released and solubilized during digestion, whereas bioavailability is reserved for absorbed material that has been demonstrated through adequate in vivo data.

### 5.2. Delivery of Hydrophilic Bioactives

Protein binding, network confinement, and aqueous phase diffusion govern the delivery of hydrophilic bioactives in GG-based systems, respectively. In simulated in vitro digestion, gelatin–GG composite hydrogels preserved anthocyanins during the stomach phase and controlled their subsequent intestine release [110]. Whey protein–GG nanocomplexes and whey protein–quercetin–GG complexes have likewise been evaluated for chemical stability, release, or in vitro bioaccessibility [114,115,116]. Unless covalent coupling is analytically confirmed, these systems are more appropriately considered non-covalent matrix modifications. These studies support formulation-dependent chemical protection and release under in vitro conditions, but they do not demonstrate improved epithelial uptake, systemic exposure, or human bioavailability.

### 5.3. Delivery of Lipophilic Bioactives

For lipophilic or mixed cargos, delivery performance depends primarily on multiphase organization, interfacial stability, and controlled disassembly of lipid-containing domains during digestion. In simulated in vitro gastrointestinal digestion, a water-in-oil-in-water emulsion embedded in an ion-set GG hydrogel co-encapsulated astaxanthin and phycocyanin and exhibited pH-responsive release [111]. WPI–LAGG microbeads provide another composite strategy, whereas conjugate-stabilized β-carotene emulsions represent direct covalent modification [19,113]. These studies establish formulation-specific loading, retention, release, or in vitro bioaccessibility; they do not by themselves establish in vivo absorption or bioavailability.

Lipophilic delivery additionally requires a digestible lipid phase and an interface compatible with lipolysis and micellarization. Strengthening a GG-containing interface may reduce storage loss yet delay the reorganization required for digestion and micelle formation. This protection–release trade-off is supported by in vitro digestion studies [19,111,117], but its physiological relevance requires confirmation in animal or human studies before conclusions can be drawn regarding absorption or systemic availability.

### 5.4. Probiotic Encapsulation and Intestinal Delivery

For probiotics, chitosan-coated alginate/GG microcapsules improved survival during storage and simulated gastrointestinal exposure under the reported formulation and strain conditions [118]. Composite hydrogels have also combined GG with other putatively prebiotic polysaccharides to couple physical protection with microbiota-related functions [119]. These are formulation-specific in vitro or storage outcomes; they do not demonstrate intestinal colonization, persistence, or clinical benefit in animals or humans. Viability should therefore be reported separately after processing, storage, gastric exposure, bile exposure, and intestinal release [120].

For probiotics, viability after processing or simulated gastric exposure is only an intermediate endpoint. A useful GG carrier must also release viable cells under intestinal conditions and preserve strain-specific function. In the absence of corresponding animal or human evidence, improved in vitro survival should be described as enhanced gastrointestinal tolerance or delivery potential rather than improved probiotic efficacy [118,119,120,121].

### 5.5. Gastrointestinal Disassembly and Nutrient Digestion

GG alteration influences digestion mostly through changes in food structure and mass transfer, rather than as an absorbed bioactive. In multicomponent foods, GG-containing structures alter the spatial organization and accessibility of substrates. GG–starch networks can influence exposure of gelatinized starch [74,122,123], protein–GG phases can alter protease access to aggregated protein domains, and GG-containing emulsions can modify the barriers between lipase, bile components, and lipid droplets [19,74,124]. Except where human evidence is explicitly identified below, these mechanistic interpretations are derived from in vitro digestion or food-structure studies and should not be generalized to human physiological outcomes.

#### 5.5.1. Starch Digestion

The starch-digestion evidence illustrates the need to distinguish polymer modification from food-matrix structuring. LAGG is produced by deacylation, but its incorporation during rice cooking does not introduce a further covalent change. Instead, ion-mediated assembly forms an LAGG-containing network within the cooked-rice matrix, which may restrict grain disintegration and starch accessibility [74,93]. The structure-based mechanism is supported by in vitro digestion and food-structure measurements [74], whereas the acute glycemic effect is supported separately by the human randomized crossover study [93].

In vitro digestion studies show that LAGG-structured rice can exhibit lower starch hydrolysis under defined formulation and processing conditions [74]. The result is consistent with restricted physical accessibility of gelatinized starch, potentially involving altered grain fragmentation, water distribution, matrix cohesion, or enzyme diffusion [74,122,123]. However, the relative contribution of these mechanisms has not been independently quantified, so reduced hydrolysis should be treated as the observed outcome and the proposed structural explanations as mechanistic interpretations.

Human evidence is currently limited to a randomized crossover study in 12 healthy adults in which one defined LAGG-structured rice formulation produced a lower acute postprandial glycemic response than the comparator rice [93]. This finding provides direct human evidence for an acute, formulation-specific response, but it does not establish that all LAGG-containing foods have a low glycemic impact or that LAGG improves long-term glycemic control. The outcome may depend on GG dose, rice variety, cooking procedure, ion environment, serving conditions, mastication, and meal composition [74,93]. No repeated-intake or long-term human evidence is available in the studies reviewed here.

#### 5.5.2. Lipid Digestion

Protein–GG Maillard conjugation provides a direct example of covalent modification at an emulsion interface. Coupling LAGG to whey protein isolate combines protein adsorption with the charge and steric contribution of the polysaccharide. The resulting conjugates produced smaller β-carotene emulsion droplets and improved resistance to pH, salt, heating, freezing, and storage [19]. These properties demonstrate more robust interfacial and bulk stabilization than the unconjugated controls under the reported conditions.

In the WPI–LAGG conjugate system, improved physical stability was accompanied by lower in vitro beta-carotene bioaccessibility [19]. Potential contributors include delayed interfacial displacement, reduced lipase accessibility, altered droplet aggregation, or restricted mixed-micelle formation, but these mechanisms were not independently resolved. HAGG-stabilized beta-carotene emulsions likewise show formulation-dependent changes during simulated digestion [14,77]. The available evidence therefore demonstrates changes in in vitro digestive transformation rather than a universal suppression of lipid digestion or a proven effect on human absorption.

#### 5.5.3. Protein Digestion

Protein digestion in the presence of GG is strongly matrix-dependent. GG may restrict protease access when it forms a continuous or slowly disassembling phase, but it may also permit or facilitate proteolysis when the composite structure is porous or readily fragmented [124,125]. In protein–GG gels, the relative timing of protein aggregation and GG gelation determines phase continuity, pore structure, water distribution, and fracture behavior [31,71], but these structural measurements do not directly establish digestibility. Direct digestive evidence is limited to formulation-specific in vitro studies. In a model pea-milk system, non-covalent GG addition did not impair gastrointestinal proteolysis and was associated with greater final proteolysis than the protein-only control under the tested in vitro conditions [124]. Accordingly, effects on human protein digestion cannot be inferred without direct animal or human evidence.

The digestive effect of Maillard-conjugated protein–GG materials is particularly uncertain. Conjugation changes accessible amino groups, protein conformation, aggregation, and steric shielding [28,35,36,126], each of which could alter protease susceptibility. Current GG conjugate studies primarily establish technological functionality and emulsion behavior [19,28,36], rather than protein digestion in animals or humans. Until comparative measurements of protein disappearance, peptide profiles, amino-acid availability, and ultimately in vivo utilization are available, these structures should be described only as having the potential to regulate protein digestion.

### 5.6. Protection–Release Trade-Offs

GG-based delivery systems can balance protection during processing or simulated gastric transit with release under simulated intestinal conditions. Gelatin–GG composite hydrogels protected anthocyanins during in vitro gastric digestion and regulated subsequent intestinal release [110]. A double-emulsion-filled GG hydrogel spatially separated astaxanthin and phycocyanin and generated pH-responsive release in simulated digestion [111], while WPI–LAGG microbeads and protein–GG nanocomplexes have been evaluated for release or in vitro availability of flaxseed bioactives, anthocyanins, and quercetin [113,114,115]. These studies demonstrate formulation-dependent retention and release, but the majority do not examine epithelial absorption, systemic exposure, or human bioavailability.

For GG-based carriers, maximum retention is not always the best outcome. A dense or persistent GG network may improve storage stability and in vitro gastric protection, but it might be detrimental if it inhibits intestinal disassembly, enzyme access, lipolysis, or cargo release in a bioaccessible state [110,111,112]. Therefore, evaluation should distinguish encapsulation efficiency, storage retention, stomach protection, intestinal release, in vitro bioaccessibility, and in vivo bioavailability as distinct goals. Claims regarding absorption or physiological benefit must be supported by animal or human evidence and should not be based solely on in vitro protection or release.

### 5.7. Colonic Fermentation

The molecular form of modified GG that enters the large intestine determines its colonic activity. Intact GG, low-molecular-weight GG, and GG-derived oligosaccharides differ in chain length, viscosity, and fermentability [16,43]. Depolymerization may reduce viscosity and network-forming ability [12,16], but it does not by itself demonstrate small-intestinal digestion, selective colonic utilization, or a physiological prebiotic effect.

For GG-derived oligosaccharides, fermentation behavior is governed by product size, linkage pattern, terminal structure, purity, and residual polymeric fractions [43,45,58]. The available evidence is based mainly on in vitro bacterial-culture or fecal-fermentation models, in which substrate degradation, microbial shifts, short-chain fatty acid production, or gas formation can be measured [43,58,59]. Such endpoints demonstrate fermentability under the tested conditions but do not establish selective utilization, gastrointestinal tolerance, or a prebiotic benefit in humans.

Current in vitro evidence indicates that GG-derived and related sphingan oligosaccharides can alter microbial growth and fermentation profiles in bacterial cultures and fecal-fermentation models [43,58,59]. Because these findings remain substrate- and model-dependent and are not supported by corresponding human intervention studies in the evidence reviewed here, GG-derived oligosaccharides are more appropriately described as prospective prebiotic substrates rather than established prebiotic ingredients. Their fermentation selectivity, effective dose, gastrointestinal tolerance, and sustained physiological effects require in vivo and human validation.

Table 3 separates observed digestive or release outcomes from evidence level and interpretation boundaries. Static or simulated in vitro digestion, in vitro fermentation, and human evidence are identified explicitly so that mechanistic proposals are not presented as established physiological outcomes.

## 6. Safety, Regulatory Assessment, and Industrial Translation of Modified and Structurally Regulated GG

Safety and regulatory assessment should begin with the identity and specifications of the final GG-derived material. Established safety evaluations apply to conventional food-grade GG that meets defined specifications, whereas oxidation, substantial depolymerization, oligosaccharide production, or covalent protein conjugation may generate products with different chemical composition, molecular-weight distribution, residual components, or digestive behavior [127,128,129]. This section therefore distinguishes established safety and regulatory information for conventional GG from the data requirements and case-specific assessment considerations applicable to structurally distinct products.

### 6.1. Safety: Established Evidence and Data Gaps

The established safety conclusion applies to conventional food-grade GG that meets the evaluated identity and purity specifications [127,128,129,130,131]. EFSA concluded in 2018 that there was no need for a numerical acceptable daily intake for gellan gum (E 418) and no safety concern at the refined exposure assessment for the reported uses and use levels [127]. The Panel also reported no concern regarding genotoxicity or carcinogenicity and considered intact GG unlikely to be absorbed [127]. However, these conclusions should not be transferred automatically to structurally distinct derivatives or fractions of GG that fall outside the assessed identity and specifications.

The magnitude and type of additional safety information required should reflect the intervention of GG. Ion, thermal, shear, and non-covalent composite structuring reorganize food structure without intentionally changing the GG backbone [1,24,61,64], so safety evaluation can focus primarily on permitted ingredients, use levels, and the final formulation. By contrast, LMW-GG or oligosaccharides change molecular-size distribution [16,43,47]; oxidation introduces new functional groups and reaction residuals [25]; and protein–GG conjugation creates a new covalent material together with Maillard reaction products [28,35,36]. These studies establish structural or technological effects, not comprehensive safety at dietary exposure.

Accordingly, safety assessment of GG should be route-specific. Depolymerized materials require molecular-weight or degree-of-polymerization profiles, gastrointestinal tolerance, and fermentation data [16,43,47,83]. Oxidized GG requires characterization of modification degree, chain scission, residual oxidant/catalyst, and low-molecular-weight reaction products [25,83]. Protein–GG conjugates require compositional characterization and consideration of digestibility and allergenicity [28,35,36,83]. Food-grade starting materials or favorable in vitro results alone do not establish safety of the reaction product at its intended dietary exposure.

### 6.2. Regulatory Status of Conventional GG and Assessment Framework for Modified Products

Current authorizations define conventional food-grade GG rather than every product produced from GG. In the United States, gellan gum is regulated under Title 21 of the Code of Federal Regulations (21 CFR § 172.665), where it is defined as a high-molecular-weight polysaccharide produced by pure-culture fermentation and may contain glyceryl and acetyl groups; the provision also specifies a residual isopropyl-alcohol limit and permits use as a stabilizer and thickener under current good manufacturing practice [128]. In the European Union, E 418 is authorized under Regulation (EC) No 1333/2008, while identity and purity specifications are set out in Commission Regulation (EU) No 231/2012, whose consolidated text is current to 18 August 2026 [129,132]. In China, permitted food-additive uses are governed by GB 2760-2024, and GB 25535-2010 remains the national food-safety standard for food-additive gellan gum [130,131]. These provisions establish the status of conventional food-grade GG; they do not by themselves classify new GG derivatives.

Whether a structurally modified GG product falls within an existing authorization cannot be determined solely from the modification method. Assessment must instead consider the identity and composition of the final material, manufacturing process, specifications and residuals, intended technological function, proposed use levels, dietary exposure, and the regulatory framework of the relevant jurisdiction [83,128,129,130,131,132]. EFSA’s 2026 guidance similarly requires characterization of identity, manufacture, composition, specifications, stability and fate in food, proposed uses, exposure, and safety data for food-additive applications [83]. Products outside the identity or specifications of authorized food-grade GG therefore require case-specific evaluation; the appropriate regulatory pathway should not be predicted from structural modification alone.

### 6.3. Industrial Translation and Feasibility Considerations

Industrial translation of GG modification and structural regulation strategies requires balancing functional performance with process scalability, reproducibility, quality control, safety assessment, and regulatory feasibility. Supramolecular regulation by ions, heat, or shear generally avoids chemical reaction and downstream purification but is highly sensitive to water mineral composition, mixing order, residence time, and thermal–shear history [17,61,62,64]. Food-matrix composite structuring also uses familiar food ingredients but remains sensitive to protein, starch, lipid, sugar, mineral, and pH interactions [11,31,32,133,134]. Chain-architecture regulation requires reproducible molecular-weight or product distributions and, depending on the route, control of thermal input, enzymes, acids, irradiation dose, or degradation products [12,43,47]. Oxidation and Maillard conjugation have the highest analytical and purification burdens because the degree of modification, residual reagents or catalysts, unreacted components, browning products, digestibility, or allergenicity may all need to be controlled [25,28,35,83].

Scale-up should thus mimic the validated structural output rather than the nominal treatment circumstances. A laboratory rheology result obtained in buffer cannot predict performance after filling, heating, freezing, drying, storage, or consumer preparation since the food matrix can change GG assembly [31,32,61,62,64]. Real-food validation should include the specific quantitative endpoint stated for the product—such as texture, syneresis, emulsion stability, print fidelity, sensory response, or digestive release—and should be carried out during a process window that accounts for batch and ingredient variability.

Industrial adoption is best justified when the chosen intervention gives a measurable advantage over a commercial GG grade or simpler formulation with comparable product quality and safety. The decision must therefore include reaction/process complexity, purification needs, scalability, reproducibility, analytical control, sensory and nutritional performance, cost, and case-specific regulatory burden. Table 1 presents these comparative differences rather than assuming that greater structural alteration always results in higher industrial value.

## 7. Challenges and Future Perspectives

The primary weakness of modified and structurally controlled GG is inconsistent evidence strength. The structural and rheological behavior of standard HAGG/LAGG and ion-regulated networks is fairly well understood, whereas direct dietary uses of oxidized, highly depolymerized, enzymatically produced, or protein-conjugated GG are significantly more limited [11,12,22,25,28,43]. Many studies provide the GG concentration or commercial acyl category without providing adequate information on molecular-weight distribution, residual acyl content, ionic composition, modification degree, or relevant thermal and shear processing parameters [16,22,64]. As a result, similar changes in viscosity, modulus, or encapsulation can arise from different mechanisms, including chain scission, helix aggregation, phase separation, or interfacial reorganization. Delivery and digestive evidence is particularly preliminary: most studies rely on model systems and in vitro digestion or fermentation, whereas direct animal and human validation is sparse [19,74,93,110,111]. Safety data for structurally distinct derivatives and industrial-scale reproducibility are also limited. These evidence gaps should be stated explicitly rather than bridged by mechanistic assumptions.

Future studies should use a minimum material-reporting framework linking GG source, acyl content, molecular-weight distribution, modification degree, ionic environment, processing history, and purification to verified chain, junction-zone, particle, interfacial, and phase structures [1,12,64,83]. Experimental designs should then connect these structural features to quantitative processing behavior, large-deformation texture, water mobility, sensory response, and, for delivery-oriented or digestion-related applications, gastrointestinal disassembly within the same food system. Mechanistic claims should distinguish observed outcomes from directly tested causal mechanisms and proposed explanations. Delivery studies should separate gastric retention, intestinal release, in vitro bioaccessibility, epithelial uptake, and in vivo bioavailability. Finally, safety and regulation should be built into the design by early consideration of residuals, digestibility, allergenicity, exposure, cost, scalability, and material identification. Such evidence-weighted development can shift GG research from general property enhancement to application-specific selection of a fixed intervention level.

## 8. Conclusions

This review distinguishes direct GG modification from structural regulation without covalent change. Covalent modification and chain-architecture regulation alter GG chemistry or molecular-size distribution, whereas supramolecular regulation reorganizes helices, junction zones, or gel particles, and food-matrix composite structuring changes phase organization through non-covalent interactions. Treating these intervention levels separately clarifies which food applications actually involve modified GG and which rely on conventional HAGG/LAGG formulation or matrix structuring.

The maturity of evidence varies across functional and translational domains. Quantitative food-structure and rheological studies provide the strongest support for gelled foods, starch/protein systems, emulsions, printing, and coatings. Delivery and digestive claims remain dominated by formulation-specific in vitro studies, with limited direct human evidence, and should not be generalized to bioavailability or long-term physiological benefits. Similarly, the established safety and regulatory status of conventional food-grade GG cannot automatically be transferred to structurally distinct derivatives. A mechanism-guided and evidence-aware strategy—selecting the least extensive structural regulation approach that achieves a defined food function while maintaining reproducibility, sensory compatibility, digestive suitability, safety, and scalability—provides a more defensible basis for future GG development.

## Figures and Tables

**Table 1:** Structural classification, translational burden, and validation level of principal gellan gum intervention routes.

Intervention Level/Route	Verified Structural Change and Food-Facing Consequence	Translation Burden	Validation Level/Evidence Boundary	Reference
Covalent modification—deacylation	Removes acetyl and L-glyceryl substituents; tighter helix packing gives firmer ion-set gels but lower elasticity.	Established industrial LAGG route; requires alkali treatment, neutralization, salt control and preservation of molecular weight.	Extensive material and food-formulation evidence; performance remains ion- and process-dependent.	[1,4,26,27,82]
Covalent modification—oxidation	Introduces carbonyl/carboxyl functionality with possible chain scission; can increase antimicrobial coating function but eliminate ion-induced gelation.	High burden: reaction control, residual oxidant/catalyst, purification and derivative characterization.	Direct food-coating evidence is limited; comprehensive safety and dietary-exposure evidence is insufficient.	[25,83]
Covalent modification—protein–GG Maillard conjugation	Covalent protein attachment; improves interfacial/technological functionality, whereas excessive reaction may impair color, flavor or digestibility.	Moderate–high burden: time/temperature/water-activity control, browning products, unreacted components and digestibility/allergenicity.	Food-model, emulsion and mayonnaise studies available; digestive evidence mainly in vitro.	[19,28,35,36,84]
Chain-architecture regulation—ultrasound depolymerization	Reduces molar mass; lowers viscosity and weakens gels while some local ordering may remain after moderate treatment.	Equipment is scalable, but acoustic/thermal dose and product distribution require control.	Strong material characterization; limited direct validation in finished foods.	[12,16,47]
Chain-architecture regulation—enzymatic/acid/irradiation-assisted cleavage	Generates shorter chains or oligosaccharide fractions; reduces entanglement/networking and enables emerging fermentation-related functions.	Route-specific enzyme/reagent/dose control and downstream separation; product heterogeneity increases QC burden.	Material and in vitro fermentation evidence; no established human prebiotic validation.	[43,45,55,56,59]
Supramolecular regulation—ions, heat, shear	Changes helix aggregation, junction-zone density or gel-particle formation without covalent change; tunes rigidity, yield stress, suspension and print recovery.	Low chemical/purification burden but high sensitivity to water minerals, addition order, cooling and shear history.	Broad model- and food-system evidence; effects remain formulation-specific.	[1,17,61,62,64,85,86,87]
Food-matrix composite structuring—proteins, starches, lipids, polysaccharides	Changes phase organization and network interpenetration; controls water partitioning, texture, interfacial stability and mass transport.	Low synthesis burden and familiar ingredients, but matrix-specific formulation and process reproducibility are complex.	Broad final-food evidence; direct digestive evidence is limited to selected systems.	[11,32,71,73,74,79,80,88,89]

**Note:** GG, gellan gum; LAGG, low-acyl gellan gum.

**Table 2:** Representative quantitative food applications of modified and regulated GG.

Modified/Regulated GG Classification	Food System and Key Conditions	Representative Quantitative Outcome	Validation Level/Evidence Limitation	References
Covalent modification—SPI–GG Maillard conjugate	Aerated gel/mayonnaise; 1.5% SPI, 300 mM CaCl_2_, 90 min heating; oil replacement 0–100%.	≈35% glycation; 20.8% higher overrun; up to 50% oil replacement retained favorable sensory acceptance.	Final-food physicochemical and sensory validation; broader digestion/safety evidence remains limited.	[28,84]
Covalent modification—oxidized GG	Active edible coating; H_2_O_2_/Cu^2+^-mediated oxidation.	Increased carboxyl functionality and antimicrobial coating activity; characteristic ion-induced gelation was lost.	Direct coating study; modification degree, residuals, migration, exposure and broader food validation remain limited.	[25]
Chain-architecture regulation—LMW-GG/GG oligosaccharides	Ultrasound, alkaline hydrolysis, enzyme, or irradiation + dilute-acid routes.	Lower molar mass/viscosity; irradiation–acid route generated oligosaccharide-rich fractions.	Predominantly material characterization and in vitro fermentation; no established final-food or human validation.	[12,43,45,47,59]
Supramolecular regulation—mixed HAGG/LAGG + Ca^2+^	Gelled foods; 0.5–1.5% total GG; HAGG/LAGG 25/75, 50/50 or 75/25; Ca^2+^ 2–80 mM.	Failure strain ≈0.6–1.5; strongest synergistic response near HAGG/LAGG = 50/50.	Model/food gel mechanical evidence; formulation-specific and not a new derivative.	[4,17,18,82,85]
Supramolecular regulation—HAGG continuous-phase structuring	O/W emulsion; 30% sunflower oil; 0.01–0.20% HAGG.	HAGG > 0.05% produced phase-stable emulsions; LAGG did not under the same preparation conditions.	Emulsion physicochemical evidence; storage stability does not establish digestive or sensory performance.	[76,77]
Supramolecular regulation—thermal GG structuring	3D-printed starch foods; 4% or 6% GG; curing transition ≈30–45 °C.	Clear post-extrusion solidification/plastic-deformation behavior; GG altered curing rate, hardness and adhesion.	Printing/rheology validation; swallowing suitability requires separate IDDSI/oral-processing evidence.	[87,90,91,92]
Food-matrix composite—LAGG–starch	Cooked rice; 1–3% LAGG during cooking.	Jasmine-rice hardness + 8–26%; chewiness ≈1.4–2.3×; predicted GI −10–22%.	Final-food physicochemical + in vitro digestion; one acute randomized crossover human study.	[29,30,74,75,93]
Food-matrix composite—GG–protein	Egg-based yogurt/pea-protein gels; yogurt 0.045–0.085% GG; pea-protein gels <0.1% to 0.4% GG.	0.045% GG: sensory score 83.57; 0.085% GG: WHC + 30.35%, hardness 10.98 → 13.76 g, adhesiveness 8.25 → 14.95 g·s; 0.4% GG formed a dense stable pea-protein gel.	Final-food/model protein-gel evidence; optimum concentration is product-specific; direct digestion evidence limited.	[31,89,94]
Food-matrix composite—LAGG–protein	Plant-protein meat analog; 2% LAGG selected after screening 0–3%; timing of addition and CaCl_2_ controlled.	GG-free anisotropy index ≤1; GG-containing systems >1 under reported conditions.	Final-food mechanical/structural evidence; processing sequence is a major determinant.	[73]
Food-matrix composite—KGM/LAGG + thymol	Blueberry coating; 0.22% thymol microcapsules + 0.075% total polysaccharides; KGM:LAGG 1:2; storage 2 ± 0.5 °C.	Shelf life 42 d (≈3× control) under reported conditions.	Commodity-specific shelf-life study; cannot be generalized across foods or storage conditions.	[60,95]

**Note:** GG, gellan gum; HAGG, high-acyl gellan gum; LAGG, low-acyl gellan gum; WPI, whey protein isolate; SPI, soy protein isolate; KGM, konjac glucomannan; IDDSI, International Dysphagia Diet Standardisation Initiative. Quantitative values are reported only when available in the cited studies and should be interpreted within the corresponding formulation, processing, and validation conditions.

**Table 3:** Digestive and release outcomes of GG-based systems with explicit evidence levels and interpretation boundaries.

Digestive Stage	GG Structure/System and Cargo	Observed Digestive/Release Outcome	Evidence Level	Interpretation/Evidence Boundary	Reference
Gastric phase	Ion-set GG hydrogels, coated particles, multiple-emulsion-filled gels, and protein–GG complexes; anthocyanins, carotenoids, polyphenols, flaxseed-derived compounds, and probiotics	Reduced gastric leakage, increased gastric retention, or improved probiotic survival, depending on formulation	Static/simulated in vitro digestion and storage studies	Formulation-specific in vitro retention or survival; does not establish subsequent intestinal absorption or in vivo bioavailability	[110,111,113,118]
Gastric-to-intestinal transition	Unmodified HAGG weak networks surrounding emulsion droplets; lipids and β-carotene	Altered droplet aggregation, emulsion transformation, and bioactive release during simulated digestion	Simulated in vitro gastrointestinal digestion	Evidence concerns non-covalently structured HAGG emulsions and should not be generalized to covalently modified GG or in vivo outcomes	[14]
Gastric and small-intestinal phases	Non-covalent protein–GG composite phases; protein	Proteolysis was retained in one pea-milk formulation under the tested conditions	Formulation-specific in vitro gastrointestinal digestion	Direct evidence is limited to one model formulation; effects on animal or human protein digestibility remain unconfirmed	[124]
Small-intestinal phase	Rice structured with an ion-set LAGG network; starch	Reduced starch hydrolysis under the tested digestion conditions	Static in vitro starch-digestion study	Supports reduced hydrolysis for the tested formulation; the human glycemic response is evaluated separately	[29,74]
	WPI–LAGG Maillard conjugate at the oil–water interface; lipids and β-carotene	Improved emulsion stability but reduced β-carotene bioaccessibility	Simulated in vitro gastrointestinal digestion	Bioaccessibility is an in vitro endpoint; causal digestive mechanisms and in vivo absorption were not established	[19]
	Ion-set GG hydrogels, coated particles, multiple-emulsion-filled gels, and protein–GG complexes; encapsulated bioactives and probiotics	Formulation-dependent intestinal disassembly, cargo release, solubilization, or microbial survival	Predominantly simulated in vitro gastrointestinal digestion	Release or in vitro bioaccessibility does not demonstrate epithelial uptake, systemic exposure, or bioavailability	[110,111,113,114,119]
Postprandial physiological response	Rice structured with an ion-set LAGG network; available carbohydrate	Lower acute postprandial glycemic response for one defined formulation	Randomized crossover human study (12 healthy adults)	Direct human evidence, but limited to an acute, formulation-specific response; no long-term glycemic conclusion	[93]
Colonic phase	Low-molecular-weight GG and structurally defined GG- or sphingan-derived oligosaccharide fractions; fermentable carbohydrates	Changes in microbial growth, substrate utilization, or fermentation products under test conditions	In vitro bacterial-culture and fecal-fermentation studies	Demonstrates fermentability in vitro; dose response, tolerance, microbial selectivity, and human prebiotic relevance remain unconfirmed	[43,58,59]

**Note:** WPI, whey protein isolate. “Bioaccessibility” denotes the fraction released and solubilized during digestion and is not equivalent to in vivo bioavailability. Evidence in this section is predominantly based on formulation-specific in vitro digestion or fermentation models. The randomized crossover rice study [93] provides the direct human evidence summarized here. No comparable animal validation was identified for most systems in Table 3; therefore, in vitro outcomes should not be generalized to animal or human physiological effects.

## Data Availability

No new data were created or analyzed in this study. Data sharing is not applicable to this article.
